# Repeated major inland retreat of Thwaites and Pine Island glaciers (West Antarctica) during the Pliocene

**DOI:** 10.1073/pnas.2508341122

**Published:** 2025-12-22

**Authors:** Keiji Horikawa, Masao Iwai, Claus-Dieter Hillenbrand, Christine S. Siddoway, Anna Ruth Halberstadt, Ellen A. Cowan, Michelle L. Penkrot, Karsten Gohl, Julia S. Wellner, Yoshihiro Asahara, Ki-Cheol Shin, Masahiro Noda, Miyu Fujimoto, Expedition 379 Science Party

**Affiliations:** ^a^Faculty of Science, Academic Assembly, University of Toyama, Toyama 930-8555, Japan; ^b^Marine Core Research Institute, Kochi University, Nangoku 783-8502, Japan; ^c^British Antarctic Survey, Cambridge CB3 0ET, United Kingdom; ^d^Geology Department, Colorado College, Colorado Springs, CO 80903; ^e^Department of Earth and Planetary Sciences, Jackson School of Geosciences, The University of Texas at Austin, Austin, TX 78712; ^f^Department of Geological and Environmental Sciences, Appalachian State University, Boone, NC 28608; ^g^Gulf Coast Repository, Scientific Ocean Drilling, Texas A&M University, College Station, TX 77845; ^h^Department of Geosciences, Alfred Wegener Institute Helmholtz–Center for Polar and Marine Research, Bremerhaven 27568, Germany; ^i^Department of Earth and Atmospheric Sciences, University of Houston, Houston, TX 77004; ^j^Department of Earth and Environmental Sciences, Graduate School of Environmental Studies, Nagoya University, Nagoya 464-8601, Japan; ^k^Research Institute for Humanity and Nature, Kyoto 603-8047, Japan; ^l^Graduate School of Science and Engineering for Education, University of Toyama, Toyama 930-8555, Japan

**Keywords:** IODP Expedition 379, Pliocene, West Antarctic Ice Sheet, Amundsen Sea, glacial history

## Abstract

The stability of the West Antarctic Ice Sheet (WAIS), crucial for predicting future sea-level rise, is threatened by ocean-forced melting in the Pacific sector of the Southern Ocean. While some geological records and ice-sheet models suggest WAIS retreat during past warm periods, reliable data constraining the extent of retreat are lacking. Detrital Nd, Sr, and Pb isotope data of sediments recently drilled at International Ocean Discovery Program (IODP) Site U1532 on the Amundsen Sea continental rise manifest repeated alternations in sediment provenance during glacial–interglacial cycles of the Pliocene (5.33 to 2.58 Mya), a time warmer than present. The variations reflect large fluctuations in WAIS extent on the Antarctic continent. A unique high Pb/low ε_Nd_ signature of sediments found at the onset of glacial intervals (3.88, 3.6, and 3.33 Ma) is attributed to the supply of detritus sourced from plutonic rocks located in the West Antarctic interior. Its isotopic signature at Site U1532 indicates major inland retreat of the WAIS during the preceding interglacials. During peak interglacials, the ice margin had retreated inland, and icebergs rafted and deposited inland-sourced detritus over 500 km across the Amundsen Sea shelf. Subsequent readvance of grounded ice then “bulldozed” these inland-derived fine-grained sediments from the shelf down to the continental slope and rise, resulting in a high Pb/low ε_Nd_ peak in the rise sediments. Our continuous Pliocene records provide conclusive evidence for at least five major inland retreat events of the WAIS, highlighting the significant vulnerability of the WAIS to ongoing warming.

## Significance

Significant melting of the Thwaites and Pine Island glaciers in the Amundsen Sea sector of the West Antarctic Ice Sheet (WAIS) threatens its stability. The WAIS state during the Pliocene, a period mirroring potential future climate warming, provides key insights into its behavior under a warmer climate. To constrain the Pliocene state of the WAIS, we analyzed geochemical provenance data on a sediment core located off the Amundsen Sea Embayment. Our reconstruction of the Pliocene configuration of the Amundsen Sea sector reveals repeated, major ice-sheet retreat events, demonstrating the WAIS’s vulnerability to warm conditions. Our findings highlight the risk of partial or even complete collapse of the WAIS under current global warming, which would cause meter-scale global sea-level rise.

**Fig. 1.**
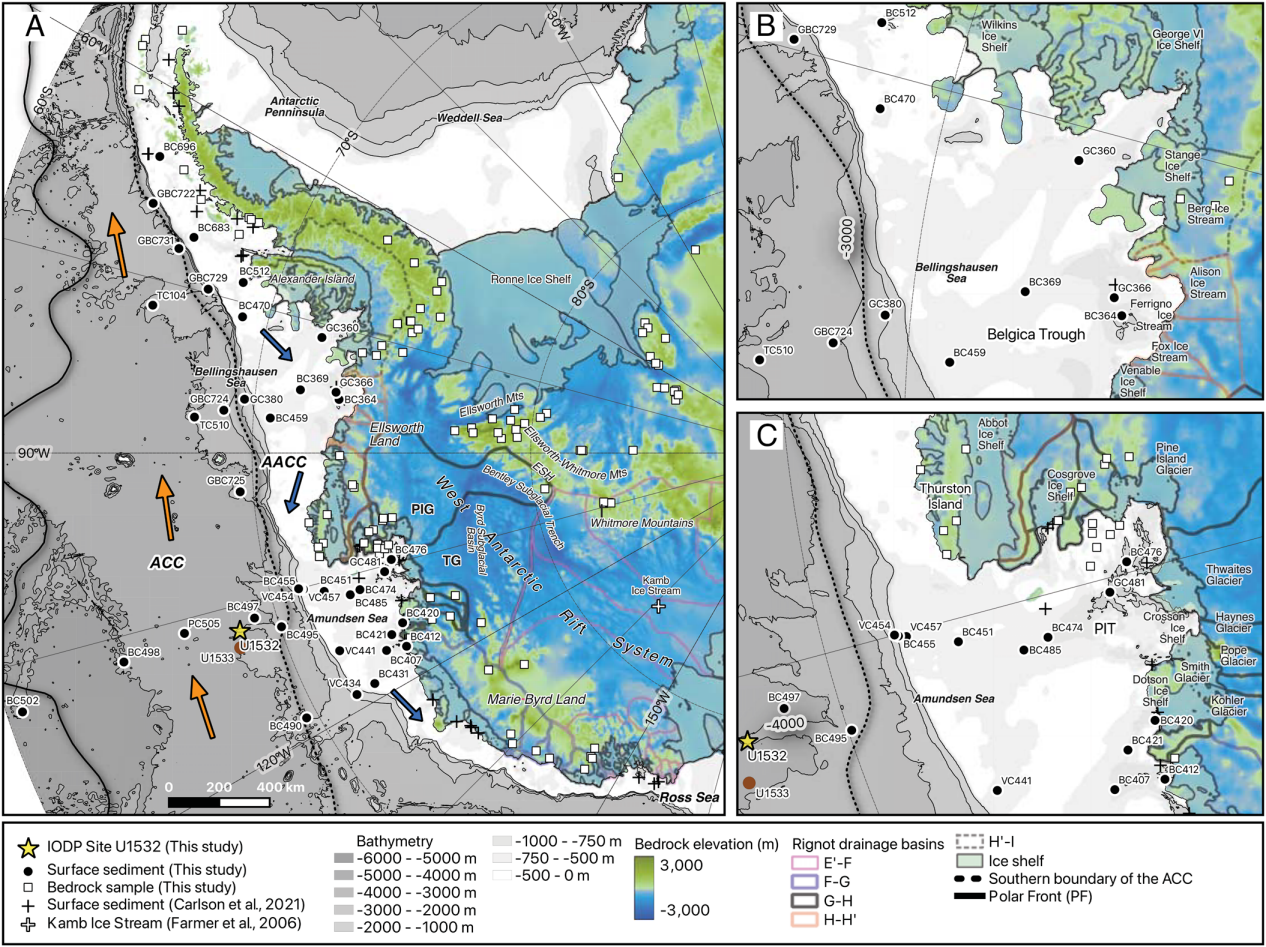
Site U1532 location and bed elevation in West Antarctica. Locations of Site U1532 (yellow star), modern seafloor surface sediment samples (black circles), and West Antarctic bedrock samples (white squares, Note that some bedrock sample sites are on islands) are shown for the entire eastern Pacific continental margin (*A*) and, in more detail, for the Bellingshausen Sea (*B*) and Amundsen Sea margins (*C*). Core sites are labeled with the core ID. The map also includes locations of samples with previously reported provenance data [filled cross symbols (10) and open cross symbol (11)], with the data shown in *SI Appendix* Fig. S5. The blue arrows mark the flow of the Antarctic Coastal Current (AACC), which flows on the shelf, and the orange arrows indicate the flow of the Antarctic Circumpolar Current (ACC), which affects Site U1532. The map was created in the Quantarctica mapping environment (12) and shows the BEDMAP2 bed elevation (5) and IBCSO seafloor bathymetry (13), together with the MEaSUREs Antarctic ice shelves, the Antarctic coastline, and subglacial Antarctic basins (14, 15). TG: Thwaites Glacier, PIG: Pine Island Glacier, ESH: Ellsworth Subglacial Highlands, PIT: Pine Island Trough. Bedrock sample ID (PRR #) is shown on a map in Fig. 3*A* and *SI Appendix* Fig. S7.

The Amundsen Sea drainage sector of the West Antarctic Ice Sheet (WAIS), particularly the catchments of the marine-terminating Thwaites Glacier and Pine Island Glacier (TG–PIG; Fig. 1*A*), is losing ice faster than other parts of Antarctica (1) due to inflow of warm Circumpolar Deep Water (CDW; ~0.5 to 1.2 °C, located below 300 to 500 m water depth) (2). The CDW melts ice shelves from below (2, 3), thus weakening their buttressing effect, and can possibly intrude far under the base of a glacier during high tides (4). The situation of the subglacial bed below sea level together with its inland sloping that exceeds 2,000 m water depth in the Byrd Subglacial Basin (5) makes grounded ice of this sector, especially TG, susceptible to run-away retreat (6, 7) (Fig. 1*A*). Ice-sheet models suggest that CDW incursion beneath TG can trigger self-sustaining WAIS recession, associated with grounding-line retreat far inland and resulting in rapid, widespread ice-sheet loss (8, 9). This scenario raises concerns about large-scale and potentially rapid, future global sea-level rise from the WAIS (9). Despite these concerns, our understanding of the response of the TG–PIG to past warming events remains incomplete based on geological evidence.

Sediment records from the Amundsen Sea spanning the Pliocene Epoch (5.33 to 2.58 Mya) may reveal a harbinger for WAIS response to future warming since global mean sea-surface temperatures (SSTs) between 4.5 Ma and 3.2 Ma were 3 to 4 °C higher than late Holocene values (16), equivalent to projected future temperature increases (17). Ice-sheet models and far-field sea-level reconstructions suggest that during the Pliocene the Antarctic Ice Sheet (AIS) and the Greenland Ice Sheet were largely reduced in extent and the global sea level was > ~15 m higher than today (18), with the melting of the AIS contributing more than 8.6 ± 2.8 m to this high-stand (9, 19). However, precisely reconstructing the AIS retreat and validating the sea-level estimates during this period are challenging. Although studies on Pliocene sedimentary sequences from the western Ross Sea shelf (20) and the East Antarctic continental rise offshore from the Wilkes Subglacial Basin (21, 22) indicate repeated AIS retreats in different subglacial basins, a complete picture remains elusive due to data limitations. Specifically, ice-proximal records (such as AND-1B) from the Ross Sea shelf are affected by hiatuses, while the more distal Wilkes Land U1361 record is affected by low sedimentation rates (2 to 3 cm/ky) and represents East AIS dynamics. Consequently, critical questions remain unanswered: Did the WAIS fully disintegrate during the Pliocene? If so, when and how often did such events occur, and what triggered them?

Here, we fill this knowledge gap by generating a high-resolution record of geochemical provenance, diatom assemblages, and element abundances (XRF core scanner data) from Pliocene marine sediments recovered on the Amundsen Sea continental rise and integrating the results with ice-sheet model simulations. The obtained data reveal fluctuations in sediment provenance that indicate repeated major ice loss from the Amundsen Sea and Bellingshausen Sea sectors of the WAIS during Pliocene interglacials. We find that, at least five times, the WAIS margin retreated far inland, to the Bentley Subglacial Trench and the Ellsworth-Whitmore Mountains block (EWM) (Fig. 1*A*).

## Results and Discussion

### Glacial–Interglacial Sediments At Site U1532.

**Fig. 2.**
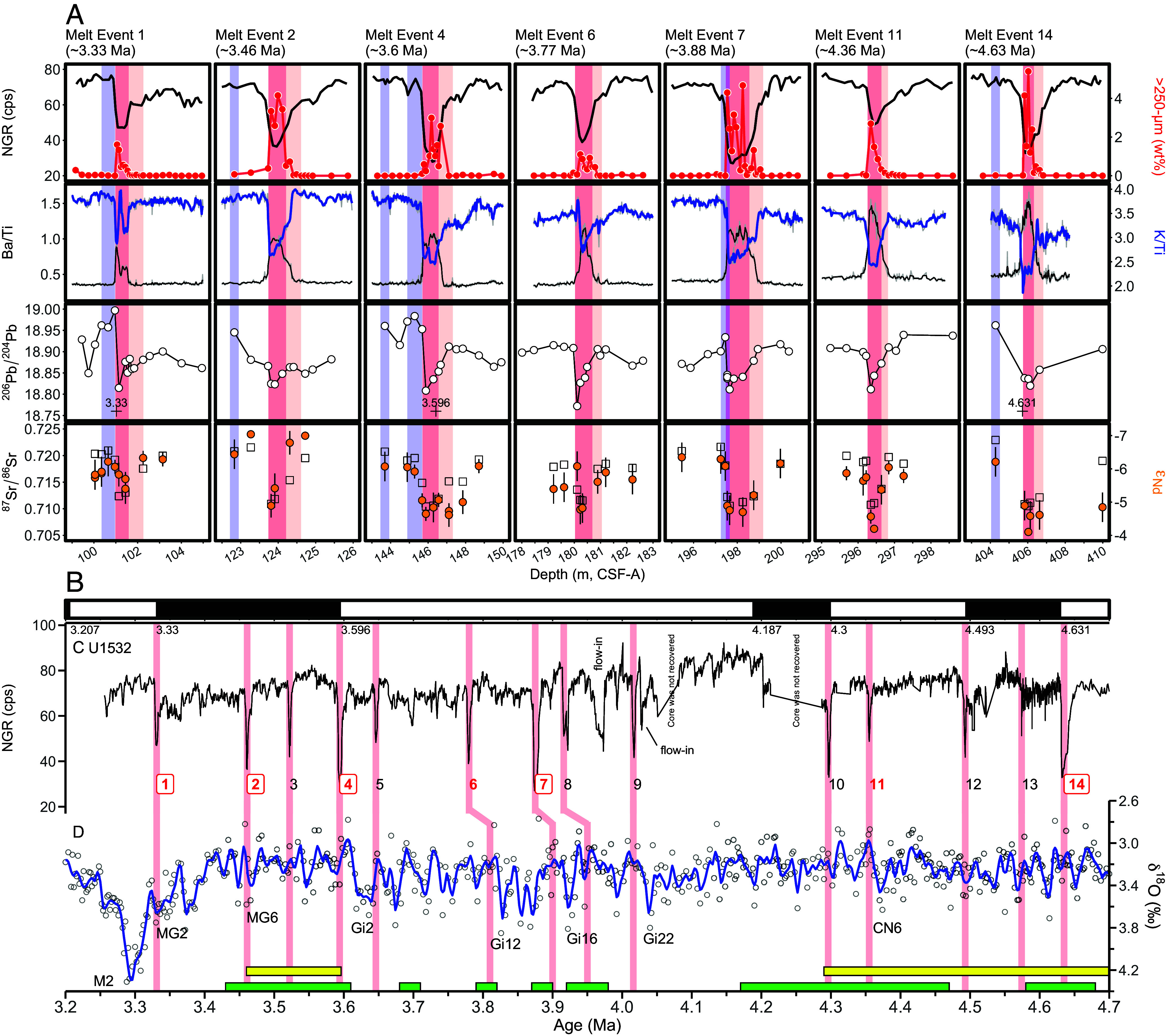
Records of ice rafting, biological productivity, and sediment provenance across seven Pliocene glacial–interglacial cycles at Site U1532. Very light red and light red shading highlight interglacial mud intervals with ice-rafted debris (IRD) and lower natural gamma ray (NGR) values, corresponding to early and peak interglacial periods. Blue shading denotes the periods showing unique high Pb/low ε_Nd_ signature of sediments during early glacial stages. Vertical shading in panels c and d show the 14 identified melt event (numbered). Numbers enclosed in squares represent major inland retreat events of the WAIS. (*A*) From top to bottom, NGR (black line), abundance of >250 µm fraction (red line), Ba/Ti (black) and K/Ti (blue) ratios (measured by XRF scanning), and ^206^Pb/^204^Pb, ^87^Sr/^86^Sr, and ε_Nd_ data of fine-grained detritus. Element ratios represent 3-point running means of 2 cm interval data points (shown by gray line). Error bars for ε_Nd_ show 2 SD external reproducibility, while errors for Sr and Pb isotopes are smaller than the symbols. (*B*) Paleomagnetic polarity data (black = normal, white = reversed) (27). Ages (Ma) of paleomagnetic reversals are also indicated. (*C*) NGR record from Site U1532. Lower NGR values indicate IRD-bearing mud intervals deposited during interglacials marked by major WAIS retreat. (*D*) Composite benthic foraminiferal δ^18^O record (28), with a LOESS smooth curve (blue line) and labels for some glacial stages. Yellow and green horizontal bars indicate periods, when the Ross Ice Shelf collapsed (20) and biological productivity offshore from the Wilkes Land margin was high (21), respectively. The ages of Melt Events 6, 7, and 8 have large uncertainties (*SI Appendix*, Fig. S2) and are tentatively correlated here with the nearest pronounced interglacials and the Wilkes Subglacial Basin melt periods.

During International Ocean Discovery Program (IODP) Expedition 379 with R/V *JOIDES Resolution*, a sediment record spanning the latest Miocene to Holocene was retrieved at Site U1532 from a sediment drift on the Amundsen Sea continental rise (23) (Fig. 1*A* and *SI Appendix*, Fig. S1 and Supporting Text). The age model for Site U1532 reveals that thick Pliocene sediments (45.41 to 580.81 m below seafloor) were deposited under medium to high sedimentation rates of 5.4 to 61 cm/ky (23, 24) (*SI Appendix*, Figs. S1 and S2). The Pliocene sediments are marked by alternations of thick, gray, predominantly terrigenous laminated silty clays with relatively thin, greenish, biosilica-bearing/rich, bioturbated muds containing dispersed iceberg-rafted debris (IRD, >250 µm), whose abundance usually increases toward the top of the muds (*SI Appendix*, Fig. S3). The IRD-bearing greenish mud intervals are typically less than 1.7 m thick and are characterized by low natural gamma ray (NGR) and magnetic susceptibility values and negative a*-values (23) (Fig. 2 and *SI Appendix*, Fig. S3). The diatom assemblages in the IRD-bearing muds are dominated by open water taxa, heavily silicified *Fragilariopsis* (*F. barronii*, *F. interfrigidaria*, and *F. praeinterfrigidaria*) and *Dactyliosolen antarcticus*, and significant biological productivity is indicated by relatively high diatom concentrations and elevated Ba/Ti ratios, which are a proxy for biogenic barium (25, 26) (Fig. 2*A* and *SI Appendix*, Figs. S3*B* and S4). The high IRD content, evidence for elevated productivity, and abundance of open ocean diatoms suggest that the IRD-bearing muds formed during interglacial periods, potentially reflecting past retreat events of the WAIS, whereas the lack of microfossils, bioturbation, and IRD in the laminated silty clays indicates their deposition under glacial conditions. In this study, we identified 14 prominent IRD-bearing mud intervals between 4.65 Ma and 3.33 Ma based on three criteria: i) NGR values decreasing below 50 counts per second (cps), ii) a concurrent decrease in magnetic susceptibility, and iii) the presence of biosilica-bearing or -rich, bioturbated muddy facies containing IRD. We interpret these prominent IRD-bearing muds as interglacial melt events (Fig. 2 and *SI Appendix*, Fig. S1).

### Provenance of Modern Seafloor Surface and Pliocene Sediments.

**Fig. 3.**
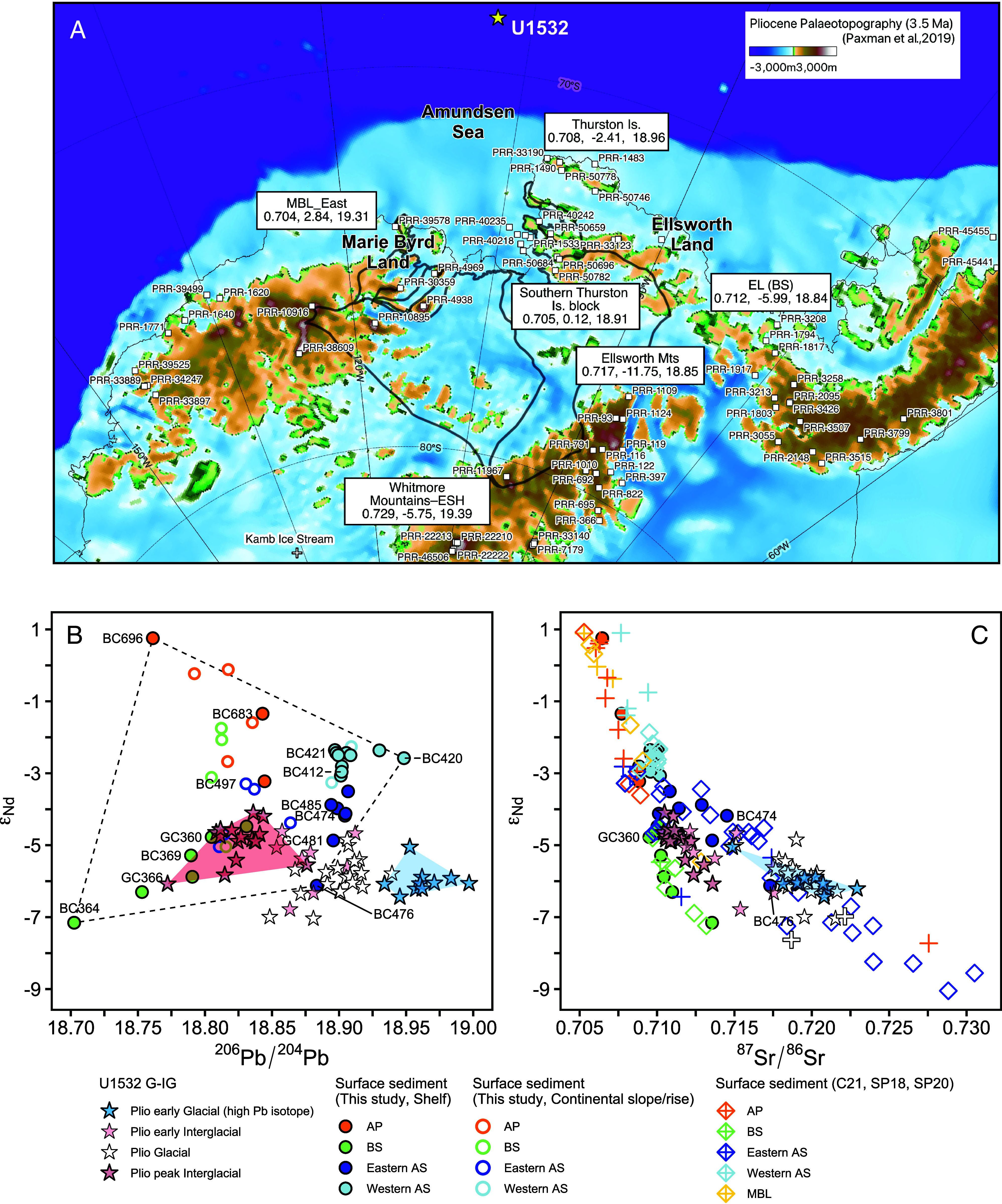
Provenance of modern seafloor surface sediments on the West Antarctic margin and Pliocene sediments at Site U1532. (*A*) Ice-free topography during the mid-Pliocene (3.5 Ma), with weighted mean isotopic compositions (^87^Sr/^86^Sr, ε_Nd_, and ^206^Pb/^204^Pb) of bedrock samples from six different West Antarctic regions (EL: Ellsworth Land; ESH: Ellsworth Subglacial Highlands; MBL: Marie Byrd Land). The estimated “median” topography during the mid-Pliocene (3.5 Ma) is shown in color (32). Elevations are given relative to present-day mean sea level and for fully isostatically relaxed ice-free conditions. Bedrock sample locations are labeled with the Polar Rock Repository identifier (PRR #). Weighted mean and SD values for each region are given in *SI Appendix*, Table S2. See *SI Appendix* Fig. S10 for all isotope data in geographic areas. (*B*) Nd versus Pb isotopic compositions of the fine-grained detritus in Pliocene samples at Site U1532 and modern seafloor surface sediments from the West Antarctic margin. Samples from shelf sites are represented by filled circles, while samples from continental slope and rise sites are shown by open circles. The provenance of the surface sediments is distinguished by different colors for the western Antarctic Peninsula (AP), Bellingshausen Sea (BS), and eastern and western Amundsen Sea (AS). Selected ice-proximal sites are labeled with the core ID. Pliocene samples from Site U1532 (stars) are divided into early and peak interglacial, glacial, and early glacial (high ^206^Pb/^204^Pb isotope ratios) samples. (*C*) Nd versus Sr isotopic compositions of the fine-grained detritus in Pliocene samples at Site U1532 and modern seafloor surface sediments from the West Antarctic margin. Data from this study, Simões Pereira et al. [SP18 and SP20, diamond (33, 34)], Carlson et al. [C21, cross (10)], and subglacial sediment underlying Kamb Ice Stream (KIS) from Farmer et al. [open cross (11)] are shown.

We analyzed strontium (Sr), neodymium (Nd), and lead (Pb) isotope ratios of fine-grained detritus (<63 µm) in the Pliocene sediments of Site U1532. These radiogenic isotopes with long-lived parents display distinct isotopic signatures that vary in rocks depending on their age, parent/daughter ratios, and initial isotopic compositions (29, 30). The isotopic compositions of the sediments help to identify source rocks and, therefore, the geographic source regions, which allows for tracing the locations of past subglacial erosion and, thus, reconstructing past WAIS extent (31). Because glaciogenic fine-grained detritus at Site U1532 may have been transported by ocean currents and icebergs over long distances (>100 km), we acquired precise isotopic fingerprints for diverse sources across a wide geographical area in West Antarctica by analyzing 42 modern, seafloor surface sediment samples covering the Pacific margin and 100 bedrock samples collected from coastal and interior outcrops (*SI Appendix*, Supporting Text). We divided this extensive bedrock dataset into eleven geographic regions (i.e., sources) and used this framework to calculate a weighted mean isotopic value of the various rock types collected within a source region, which allowed us to provide an integrated isotopic signal for each source region (Fig. 3*A* and *SI Appendix*, Table S2 and Fig. S10).

Surface sediment samples from the inner continental shelf contain detritus eroded and supplied by nearby glaciers. Near-coastal samples from the western Antarctic Peninsula shelf (BC696 and BC683), Bellingshausen Sea (BC364 and GC366), and eastern (BC476) and western Amundsen Sea (BC420, BC421, and BC412) display unique and very different Sr-Nd-Pb isotopic compositions (Fig. 3 *B* and C and *SI Appendix*, Fig. S5). This difference stands out in the ε_Nd_ versus ^206^Pb/^204^Pb isotope plot, where the samples from the various coastal regions form distinct end members (Fig. 3*B*). The isotopic compositions of modern sediments from the middle and outer shelf, slope and rise plot between these end members, suggesting that offshore sediments consist of a mixture of detritus derived from four end-member regions in varying proportions, without any significant contribution from the Ross Sea sector (*SI Appendix*, Fig. S5). In the Amundsen Sea embayment (ASE), PIG-sourced detritus (–9 ε_Nd_) (33) is today rapidly diluted northward across the shelf by detritus supplied from the Thurston Island block and transported westward by the Antarctic Coastal Current (AACC), so that the PIG signal is hardly detectable in sediments on the middle to outer shelf (e.g., –4 ε_Nd_ at site BC485) (33, 34). This dilution process weakens the distinct, individual provenance signals from both TG and PIG, making the Thurston Island block the dominant source for sediments deposited on the middle to outer shelf today. At continental rise sites, like BC497 near Site U1532, the eastward-flowing Antarctic Circumpolar Current (ACC) (35) influences fine-grained sediment transport and its Sr-Nd-Pb isotopic compositions. In addition, other ocean currents, including the westward-flowing AACC and downslope and bottom currents along the continental margin, also exert secondary effects on spatial distribution of Sr-Nd-Pb isotope data of fine-grained sediments (24, 34) (Figs. 1*A* and 3*B*). These seafloor surface sediment data suggest that down-core changes in the isotopic compositions of Pliocene sediments at Site U1532 likely reflect a combined influence of the past variations in the ocean current system and supply of detritus from source regions, linked to the extent of the WAIS (26).

To reconstruct past WAIS extent during the Pliocene, detailed sediment provenance analyses were conducted on samples from seven glacial–interglacial (G–IG) cycles between 4.65 Ma and 3.33 Ma, which include distinct interglacial melting events of the WAIS marked by IRD-bearing greenish muds (Fig. 2). These seven G–IG cycles, selected from fourteen melting events identified within this period, were chosen for our detailed investigation because they were recovered as an exceptionally well-preserved sedimentary sequence in a single 10 m-long core, with some of them being dated directly, containing pristine diatoms, and being characterized by IRD-bearing horizons of various thickness (Fig. 2 and *SI Appendix*, Figs. S3 and S4). This combination of factors allows us to better understand the WAIS’s dynamic response to Pliocene warmth, as influenced by varying climate conditions. Additionally, ice-sheet retreat occurred in both the Ross Sea Embayment (20) and on East Antarctica’s Wilkes Land margin (21, 22, 36), when the seven “melt events” in the Amundsen Sea sector occurred (Fig. 2*D*). Furthermore, SSTs were slightly elevated in the SW Pacific (37, 38) (*SI Appendix*, Fig. S6), mean annual SSTs were up to 4.5 to 5.5 °C warmer south of the Antarctic Polar Front (APF) (39), Antarctic sea-ice extent was at a minimum (40), and the APF shifted significantly southward (41) during this time.

We divide each G–IG cycle into four distinct stages: a glacial, early interglacial, peak interglacial, and glacial-onset stage (*SI Appendix*, Fig. S3). The first three stages are characterized by distinct IRD content, diatom abundance/assemblage, Ba/Ti (biological productivity), and K/Ti (sediment provenance) ratios, as well as Sr-Nd-Pb isotope ratios. The glacial-onset stage is specifically identified by an interior West Antarctic provenance signal, as evidenced by detrital Nd and Pb isotope data discussed later.

Figs. 2*A* and 3*B* show that the Sr-Nd-Pb isotope ratios from the seven G–IG cycles display distinct variations, with the provenance of glacial silty clay and interglacial mud forming two separate clusters in the ε_Nd_–^206^Pb/^204^Pb isotope plot. Pliocene glacial sediments exhibit a close isotopic resemblance to modern sediments at site BC476 in Pine Island Bay (PIB), i.e., proximal to the TG and PIG termini (Fig. 3 *B* and *C*). Seismic data show multiple buried grounding zone wedges preserved within the Pliocene sequence on the outer Amundsen Sea shelf, indicating repeated advances of a grounded ice-stream from TG and PIG, followed by long periods of glacial retreat (24). The WAIS configuration during glacials, with its margin grounded on the outer shelf, would have facilitated abundant downslope sediment transport to the rise (42), and thus, explains the observed dominant supply of PIB-derived detritus to Site U1532 during each glacial.

Sediment provenance shows subtle variations between early and peak IG stages, reflecting the varying WAIS extent during interglacial periods (Fig. 2*A*). The early IG stages are marked by the onset of bioturbation (23) and a slight increase in coarse-grained detritus (>250 µm, <0.5 wt%) interpreted as IRD, with a subsequent increase in marine productivity (Ba/Ti) and a decrease in the K/Ti ratio (Fig. 2*A* and *SI Appendix*, Fig. S3). Concurrent with these changes, the detrital Nd and Pb isotopic compositions are slightly shifted toward the provenance of modern sediments in Pine Island Trough (sites GC481, BC474, and BC485; Figs. 1*C* and 3*B*). The igneous rocks of Thurston Island (43–45) and the Thurston Island block further south, located to the east of Pine Island Trough, are potential sources for early IG sediments deposited at Site U1532. The bedrock of this region consists of granite, diorite, gabbro, basalt, and hyaloclastite with ε_Nd_ values higher than –5. Some of these rocks (e.g., PRR-33190, -50746, -1483, -40235, and -40242) also exhibit low K/Ti and low ^206^Pb/^204^Pb ratios (*SI Appendix*, Fig. S7). This bedrock composition accounts for the observed geochemical signatures of the early IG sediments. While the bedrock in eastern Marie Byrd Land (MBL) has similar K/Ti ratios and ε_Nd_ values, it is distinct from the Thurston Island block rocks by higher ^206^Pb/^204^Pb ratios (*SI Appendix*, Fig. S7). Therefore, we attributed the geochemical signature of the early IG rise sediments to an increased contribution of detritus from Thurston Island and the Thurston Island block further south, driven by the melting of the grounded ice stream from these areas.

During peak IG stages, a significant IRD increase (up to 5.4 wt%) is observed, suggesting increased supply and/or melting of icebergs. Simultaneously, Ba/Ti and K/Ti ratios reached their peak and stabilized (Fig. 2*A* and *SI Appendix*, Fig. S3). In the Nd-Pb isotope space, fine-grained detritus at Site U1532 plots in an area between material derived from the Bellingshausen Sea coast (GC360 and GC366) and the eastern Amundsen Sea coast (BC485) (Fig. 3 *B* and *C*). Crucially, the U1532 peak IG sediments also have Sr-Nd-Pb isotopic compositions that closely match the weighted mean of bedrock samples from Ellsworth Land (Fig. 3*A*). Moreover, some rock types, represented by PRR-3426 and -3213 from the EL (BS) sector, exhibit less radiogenic Pb isotopic ratios (18.73 to 18.77 for ^206^Pb/^204^Pb) and low ε_Nd_ values (–9.6 to –7.5) that account for the isotopic features of the peak IG sediments (*SI Appendix*, Figs. S7 and S8). These relationships strongly support the interpretation that a large amount of detritus from this inland region was delivered to Site U1532 during peak IGs, a conclusion consistent with that based on recently published ^40^Ar/^39^Ar dates of early Pliocene IRD from sites U1532 and U1533 (46). In summary, our isotopic analyses of the seven prominent IG intervals suggest that distinguishable differences in sediment provenance correspond to sequential reduction in ice-sheet extent from glacial to early interglacial and then to peak interglacial stages along coastal West Antarctica.

The return to glacial conditions is marked by an abrupt cessation of IRD deposition and reappearance of typical glacial sediment facies (23) at Site U1532 (*SI Appendix*, Fig. S3). This facies resulted from the WAIS readvance across the Amundsen Sea shelf and long-lasting sea-ice cover, similar to conditions of formation of Quaternary glacial sediment facies on various parts of the Antarctic continental margins (47). This is consistent with periods of prograding sequence build-up on the outer shelf and slope (24). Within sediment deposited at the onset of three glacial intervals (3.88 Ma, 3.6 Ma, and 3.33 Ma), a distinct, sharp ε_Nd_–^206^Pb/^204^Pb signature appears, marked by ε_Nd_ below –5 and ^206^Pb/^204^Pb higher than 18.93, and elevated ^207^Pb/^204^Pb and ^208^Pb/^204^Pb ratios (Fig. 3*B* and *SI Appendix*, Figs. S8 and S9). The high Pb isotope signatures observed during the early glacial intervals cannot be explained by a mixture of detritus supplied from coastal West Antarctica, such as the Antarctic Peninsula or the areas directly landward of the Bellingshausen and Amundsen Sea embayments (Fig. 3*B*). Instead, on a ^206^Pb/^204^Pb–^207^Pb/^204^Pb plot (*SI Appendix*, Fig. S8), the data from the early glacial sediments form a linear array that intersects the granite field of the Whitmore Mountains and Ellsworth Subglacial Highlands (ESH), located in the West Antarctic interior. This intersection occurs at a ^206^Pb/^204^Pb value of ~19.4, indicating that the Whitmore Mountains–ESH granites act as the key radiogenic end-member and were a greater source component in the early glacial sediments. Indeed, the weighted mean isotopic compositions of bedrock samples from these areas (n = 11) have the necessary high ^206^Pb/^204^Pb ratios (19.39 ± 0.47) and low ε_Nd_ values (–5.75 ± 2.0) (*SI Appendix*, Table S2). Thus, this unique high Pb/low ε_Nd_ signature, observed at the onset of specific glacial periods, can only be explained by the delivery of detritus eroded from the Whitmore Mountains–ESH (Fig. 1*A*).

**Fig. 4.**
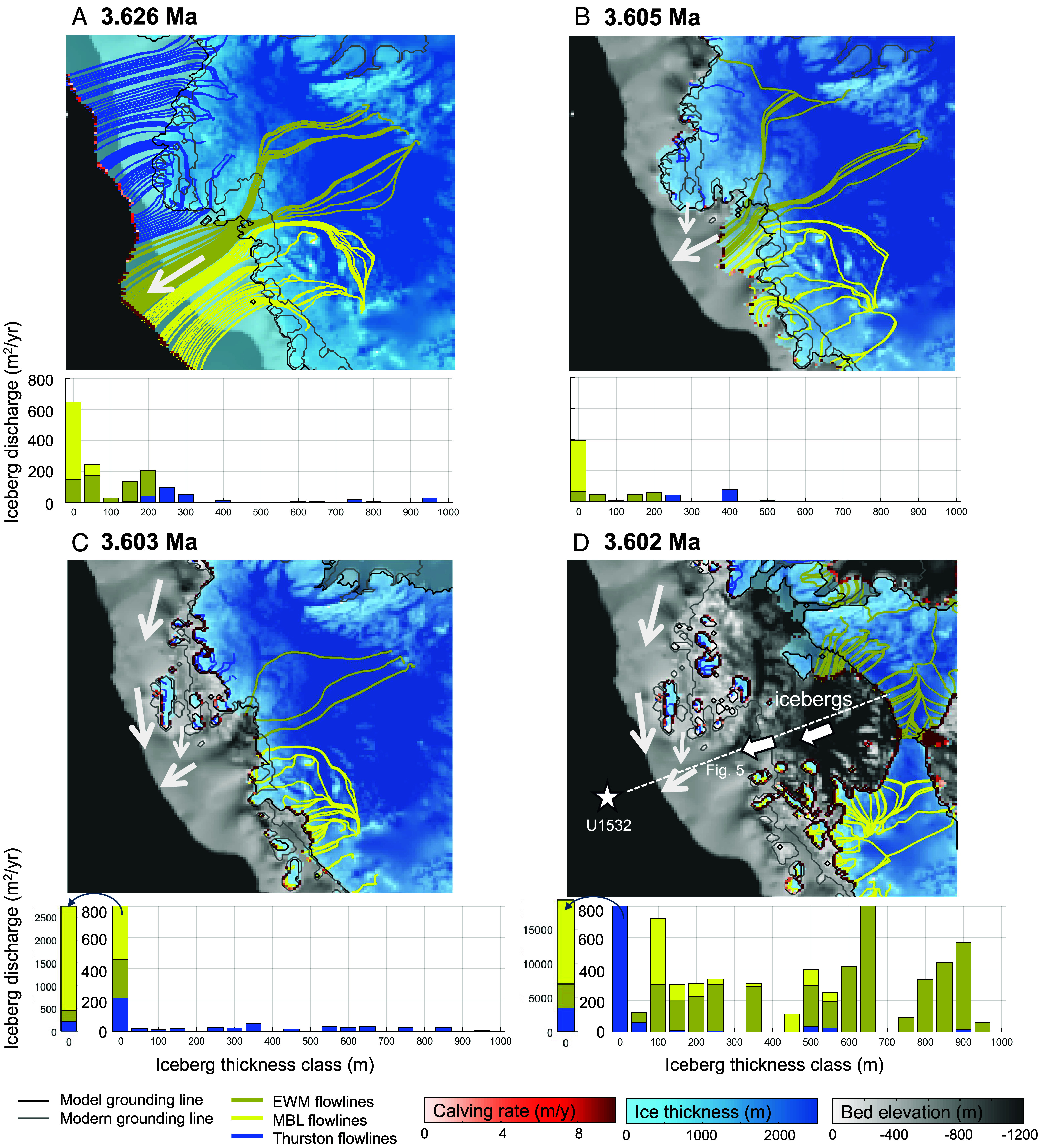
Simulated retreat stages of the WAIS at 3.6 Ma. Model snapshots of WAIS retreat, from a transient 10 km resolution nested simulation (50). The snapshots depict three key phases: (*A*) maximum ice-sheet extent phase, (*B* and *C*) early interglacial phase: The ice cap over Thurston Island begins to shrink and grounding line along Antarctica’s coast retreats inland, and (*D*) peak interglacial phase: The TG–PIG ice margin retreats into deep basins below sea level. The WAIS could have calved up to >500 m thick icebergs near the EWM during this phase. Flow lines offshore from the paleo-grounding line represent ice-shelf flow. The white arrows depict pathways of detritus transport to Site U1532 inferred from sediment isotope data. Delivery of basal ice–entrained debris from each of these sources is represented by combining model calving locations and rates with model ice flowlines. Basal ice flowlines are plotted only, if they are sourced from a targeted region (EWM, MBL, and Thurston Island/Ellsworth Land, as designated in Polar Rock Repository collection) and terminate at a grid cell where calving is occurring. Model calving rate is plotted at the calving line. Areal ice discharge is calculated for each iceberg size fraction by multiplying the number of calving grid cells with ice thickness, multiplied by the calving rate. Discharge of the smallest iceberg class is truncated at 800 m^2^/y in the main plots of panels (*C* and *D*), with the full discharge rate additionally displayed to the *Left* of the main plots (arrows). The dash line in Fig. 4*D* indicates the cross-section shown in Fig. 5. TG–PIG: Thwaites Glacier–Pine Island Glacier, EWM: Ellsworth-Whitmore Mountains, MBL: Marie Byrd Land.

Modeled ice-sheet trajectories indicate that debris eroded in the Whitmore Mountains–ESH can be transported toward both the Ross Sea and the Amundsen Sea embayments during times of major WAIS retreat (Fig. 4*D*). To ensure that our limited Whitmore Mountains–ESH bedrock samples provide a representative isotopic reference, we compared them with subglacial sediments from the Kamb Ice Stream (KIS) that drains into the Ross Ice Shelf downstream of the Whitmore Mountains–ESH (11) (Fig. 1*A*). Modern till underlying the KIS exhibits a provenance signature characterized by a high ^206^Pb/^204^Pb ratio (19.093) and a low ε_Nd_ value (–7.31), consistent with that observed for subglacial sediments underlying the Whillans and Bindschadler ice streams and sub-ice shelf sediments recovered by the Ross Ice Shelf Project (RISP) (11) (*SI Appendix*, Fig. S11). The isotopic composition of these sediments is explained by the mixing of detritus derived from Paleozoic metasedimentary rocks, similar to those exposed in westernmost MBL (and Sulzberger Bay sediments, *SI Appendix*, Fig. S9), with that from Permian‐Early Jurassic rocks in the West Antarctic interior (11, 48). Therefore, we conclude that high ^206^Pb/^204^Pb ratios (19.39 ± 0.47) and low ε_Nd_ values (–5.75 ± 2.0) of Whitmore Mountains–ESH bedrock samples provide a reasonable estimate for the isotopic end-member of the West Antarctic interior. The high Pb/low ε_Nd_ signature at Site U1532 can be attributed to detritus derived from exposed and subglacial bedrock of the Whitmore Mountains–ESH (49).

### WAIS Dynamics During the Pliocene Glacial–Interglacial Cycles.

To explore whether the distinguishable differences in geochemical sediment provenance characterizing the seven prominent IG intervals and the onset of glacial intervals at Site U1532 really reflect sequential reduction in the extent of the WAIS and its major inland retreat, we consider them in the context of time-evolving ice-sheet model simulations of G–IG retreat and readvance of the WAIS under Pliocene boundary conditions (50). Here, we select the best-fit multimillion-year continental simulation, validated by a suite of ice-proximal geological data, and downscale over the Amundsen Sea region to investigate WAIS dynamics at a higher spatial and temporal model resolution (10 km, with model output every 100 y).

Fig. 4 shows model snapshots of WAIS retreat at ~3.6 Ma (i.e., Melt Event 4 in Fig. 2). The model shows that the WAIS reached its greatest extent over the ASE inner shelf at ca. 3.626 Ma, with grounded ice having advanced across the inner shelf, including PIB, and an ice shelf extending seaward to the shelf break (Fig. 4*A*). By ca. 3.605 Ma, the modeled ice-sheet configuration transitioned to a state more comparable to the modern IG period (Fig. 4*B*), and over the next 2,000 y, the ice cap over Thurston Island shrinks significantly, followed by the complete melting of the Cosgrove and the Abbot ice shelves (Fig. 4*C*). Following the disappearance of these ice shelves, the TG–PIG ice margin rapidly retreats into deep marine basins (Fig. 4*D*) within a timeframe of <1,000 y, a rate which has also been suggested by previous studies (6, 8, 19).

**Fig. 5.**
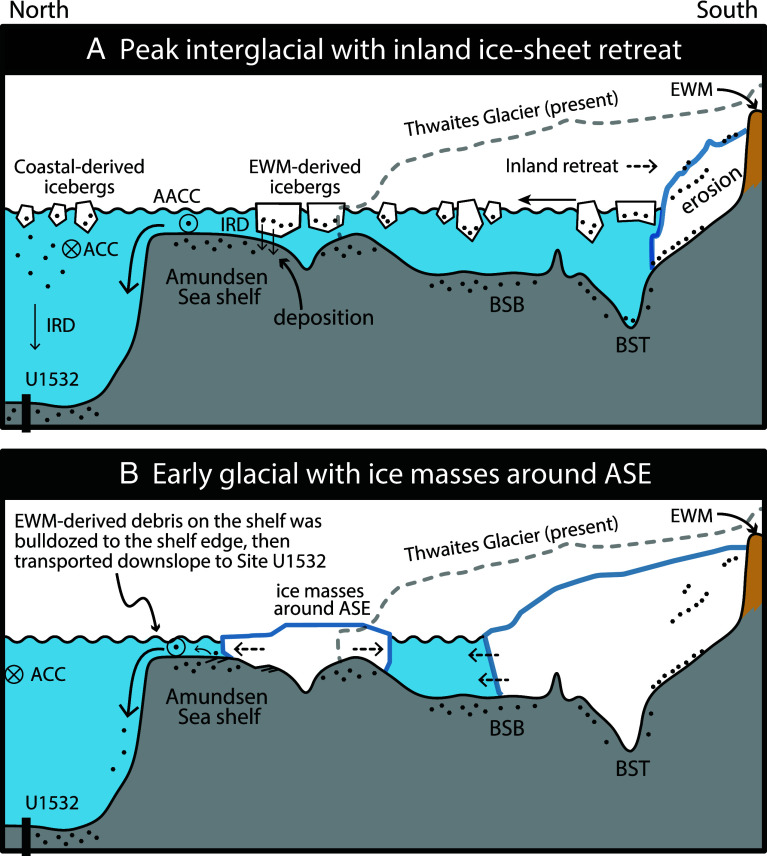
Conceptual models of ice-sheet extent and associated depositional processes along a transect (shown in Fig. 4*D*) from Site U1532 to the EWM (horizontal axis not to scale). (*A*) During peak interglacials with inland ice-sheet retreat, EWM-derived icebergs calve from the retreating ice margin and drift onto the Amundsen Sea shelf. As these icebergs ground and melt, they release most of their EWM-derived detritus on the shelf. (*B*) During early glacial stages, the ice masses around the ASE could regrow rapidly and coalesce into an ice sheet. The advancing grounded ice “bulldozed” the sediments, which had accumulated on the shelf during the previous interglacial and contain vast amounts of EWM-derived IRD, toward the shelf break. From there, the fine-grained detritus is transported down to the continental slope and rise mainly by gravitational processes, causing the high Pb/low ε_Nd_ signal in the early glacial sediments deposited at Site U1532. BSB: Byrd Subglacial Basin, BST: Bentley Subglacial Trench.

When the WAIS grounding line retreated during the IG period from 3.626 Ma to 3.602 Ma (Fig. 4), sediment-laden meltwater plumes generated at the receding ice margin probably were deposited as plumites on the Amundsen–Bellingshausen Sea shelves (51). The fine-grained particles of these plumes were advected westward by the AACC and also transported further offshore by ocean currents and gravitational downslope processes, with the latter supplying them via gullies on the continental slope and deep-sea channels on the rise to Site U1532 (42). The WAIS configuration in the simulations (Fig. 4 *B*–*D*) and the inferred ocean circulation patterns are consistent with detrital isotopic signals from Thurston Island and the Bellingshausen Sea sediments that are prevalent in the IG sediments at Site U1532 (Fig. 3). The reduced contributions of detritus from the PIB region to the U1532 sediments during all peak IG stages can be explained by the retreat of the TG–PIG terminus far inland of today’s ice front (Figs. 4*D* and 5*A*). This significant retreat shifted the dominant sediment supply to detritus originating from the Bellingshausen Sea, transported by the AACC (Fig. 3). The major upstream retreat of TG–PIG is also substantiated by the sudden appearance of reworked middle Miocene diatoms (<3%) observed in the IG sediments at Site U1532 (at 4.63 Ma, 3.88 Ma, 3.6 Ma, and 3.33 Ma) (*SI Appendix*, Fig. S4). During these peak IG stages, subglacial reworking of diatoms from middle Miocene sediments on the ASE shelf is considered unlikely because the entire Miocene sequence is relatively thin and can only be eroded on the ASE mid-shelf (24). More plausible sources of middle Miocene diatoms are subglacial strata in the Siple Coast drainage basin, Byrd Subglacial Basin, and Bentley Subglacial Trench (Fig. 1*A*), as Miocene-dominated marine diatom assemblages have been reported from modern subglacial sediments recovered along the Siple Coast close to the Transantarctic Mountains (52). Therefore, we argue that the reworked middle Miocene diatoms were actively eroded and transported offshore when the WAIS retreated upstream toward the EWM (Fig. 4*D*).

A crucial finding from isotopic analyses of Site U1532 sediments is the high Pb/low ε_Nd_ signature at the onset of specific glacial periods (3.88 Ma, 3.6 Ma, and 3.33 Ma). This signal is diagnostic of plutonic sources in the Whitmore Mountains and the ESH sectors of EWM, and detritus containing this fingerprint at Site U1532 requires WAIS retreat far inland during the immediately preceding IG. Our ice-sheet model simulations of such extensive WAIS retreat involve very high calving rates of very thick icebergs (>500 m) from the receded ice margin (Fig. 4*D*). Icebergs calved from glaciers flowing through deep valleys in the EWM and draining the ice-sheet remnant there likely contained abundant englacial and supraglacial debris in addition to subglacial debris (53). The icebergs initially drifted over the deep, seawater filled Bentley Subglacial Trench and then, driven by intensified ocean currents and winds that affected the newly opened seaways between the Amundsen, Bellingshausen, and Weddell Sea embayments, onward across the ASE shelf (Fig. 4*D*). There, these thick icebergs are likely to have run aground on the relatively shallow ASE shelf, which during Pliocene IGs was probably even shallower than today because of less overdeepening by previous subglacial erosion (32) and glacio-isostatic rebound in response to WAIS loss. Consequently, the icebergs could have released most of their EWM-derived detritus on the shelf as they melted, spreading a late-IG veneer of sediment with the unique high Pb/low ε_Nd_ signature across the ASE shelf (Fig. 5*A*).

Although this grounding process is supported by evidence from the last deglaciation (54), the initial dimensions of calved icebergs and their survival during drift are key to their ability to ground on the shelf without capsizing. Established theory indicates that an iceberg with an aspect ratio *ε* = *W*/*H* (where *W* is width and *H* is thickness) exceeding ~0.75 is hydrostatically stable and will not capsize without a significant energy input to overcome this stability (54, 55). Modern observations of the Pine Island and Thwaites ice streams show a characteristic crevasse spacing of about 1 km or more, which dictates the width of calving icebergs (54). If we assume Pliocene crevasse spacing on the retreating WAIS was comparable to today, then the resulting wide icebergs would have a high degree of initial stability (*ε* > 0.75, assuming thickness of 500 to 900 m). This stability would have allowed them to ground on the ASE shelf, while small icebergs likely passed through to the open ocean (Fig. 5*A*). A retreating WAIS could also have released meltwater plumes containing EWM-sourced fine-grained detritus from its grounding line into the Byrd Subglacial Basin, but this process rarely transports fine-grained detritus over several hundred kilometers. Therefore, we conclude that icebergs were the sole mode of transport capable of delivering fine-grained detritus with the isotopic signature of the West Antarctic interior to the ASE shelf during times of major WAIS retreat.

The EWM isotopic signature is not detected at continental rise Site U1532 during peak IGs. This suggests that the overwhelming supply of fine-grained detritus and IRD from the coastal Bellingshausen and eastern Amundsen Sea regions (46) would have likely suppressed the inland isotopic signature below the detection limit (Fig. 5*A*). Conversely, the EWM isotopic signature appears during the subsequent transition into an early glacial stage. The model simulation shows the WAIS to disintegrate into several alpine ice caps upon the elevated terrain of MBL, Ellsworth Land, and Thurston Island (Fig. 4*D*), as predicted by other ice-sheet models (19, 56). Once the climate cooled again, the ice masses around the ASE could regrow rapidly and coalesce into an ice sheet (57). Although the modeled ice-sheet grounding line only reaches a mid-shelf position during this time, the limited advance in the model could be attributed to uncertainties in key boundary conditions, such as paleo-shelf bathymetry (i.e., depth and width) and the time-evolving CO_2_ forcings (50). We envision that during the early glacial stage, advance of grounded ice near the ASE across the shelf “bulldozed” the EWM-derived debris to the shelf break, thereby mixing it with PIB-sourced detritus (BC476). From there, the material was transported downslope to the continental rise, where it created the high Pb/low ε_Nd_ signal in the sediments deposited during glacial onsets at Site U1532 (Fig. 5*B*).

An alternative explanation for the low ε_Nd_ signal (–6 ε_Nd_) in early glacial sediments could theoretically be a reorganization of the PIG and TG ice streams. However, a simple two-component mixing model of ε_Nd_ and ^206^Pb/^204^Pb between PIG (–6.1 for ε_Nd_ and 18.88 for ^206^Pb/^204^Pb at BC476) and TG (–5.4 for ε_Nd_ and 18.92 for ^206^Pb/^204^Pb at NBP99-02-49TC (10)) material cannot account for the distinctly high ^206^Pb/^204^Pb ratios (18.93 to 19.0) and low ε_Nd_ (–6 ε_Nd_) isotope signature we observe in early glacial sediments (Fig. 3*B*). This discrepancy is resolved by the introduction of a Whitmore Mountains–ESH component distinguished by high ^206^Pb/^204^Pb ratios. A simple binary mixing model, using the isotopic signatures of sample BC476 (–6.12 ε_Nd_, ^206^Pb/^204^Pb = 18.883, 38.9 µg/g [Nd], and 30.6 µg/g [Pb]) and the weighted mean of Whitmore Mountains–ESH bedrock samples (–5.75 ε_Nd_, ^206^Pb/^204^Pb = 19.386, 30 µg/g [Nd], and 22 µg/g [Pb]) as end-members, indicates that up to 30% of the glacial-onset signal at Site U1532 may originate from the Whitmore Mountains–ESH (*SI Appendix*, Fig. S11). This fact strongly supports the interpretation that the sudden appearance of provenance signal from the West Antarctic interior in early glacial sediments on the continental rise entails sediment remobilization by the advancing ice sheet across the ASE shelf.

### WAIS Destabilization and Implications for the Future.

The distinct high Pb/low ε_Nd_ signature found at the onset of Pliocene glacial periods provides evidence for major inland retreat of the WAIS toward the Bentley Subglacial Trench during preceding IGs, at least at 3.33 Ma, 3.6 Ma, and 3.88 Ma (Fig. 2*A*). Similar provenance signatures at 3.46 Ma and 4.63 Ma also suggest potential WAIS retreat far inland, albeit this is based on single data points only. The presence of reworked middle Miocene diatoms at 4.63 Ma, 3.88 Ma, 3.6 Ma, and 3.33 Ma independently supports the possibility of WAIS retreat upstream toward the EWM (*SI Appendix*, Fig. S4). Conversely, the absence of the EWM-derived isotopic signal during G–IG cycles at 3.77 Ma and 4.36 Ma and the limited number of melt events (14 times) at Site U1532 between 4.65 Ma and 3.33 Ma when compared to the more frequent interglacial peaks in the global deep-sea benthic foraminiferal δ^18^O record (28), indicate that the WAIS did not always fully disintegrate during Pliocene IGs, despite slightly elevated SSTs in the SW Pacific (37, 38) throughout the Pliocene (*SI Appendix*, Fig. S6). In summary, our data and model results suggest that the Amundsen Sea drainage sector of the WAIS maintained a persistent presence on the ASE shelf, punctuated by occasional rapid retreat events that forced ice margin recession into the Byrd Subglacial Basin or even further inland, rather than experiencing permanent retreat throughout the Pliocene (24).

Major WAIS retreat is often attributed to high atmospheric CO_2_ concentration (400 ppm) and elevated insolation levels (9, 19, 20, 36, 50). However, some Pliocene *p*CO_2_ records also imply that atmospheric *p*CO_2_ levels possibly remained below 350 to 400 ppm (58, 59). Furthermore, while some well-dated melt events (events 1, 10, and 14) coincide with relatively higher summer insolation at 80ºS, melt event 4 at 3.6 Ma, which was characterized by a full WAIS collapse, occurred under a relatively lower insolation condition (*SI Appendix*, Fig. S6). This discrepancy suggests that WAIS retreat may have been additionally influenced by intrinsic factors beyond radiative CO_2_ forcing (i.e., increased air and seawater temperatures) and insolation.

Our observations indicate that WAIS retreat often coincided with the termination of pronounced glacial periods, such as at 3.33 Ma, 3.6 Ma, and 3.88 Ma, respectively (Fig. 2). While the ice-sheet model simulations do not show grounded ice advance to the ASE outer shelf (Fig. 4*A*), this is likely because the model does not incorporate glacial sea-level lowering at 3.626 Ma. We envision that lower sea levels during the glacial periods promoted episodic grounded WAIS advances toward the shelf break, with the grounding-zone wedges observed on the outer shelf (24), possibly marking the maximum advance positions of the grounding line rather than being retreat features. These advances carved deeper and wider subglacial troughs into the Amundsen and Bellingshausen Sea shelves, as evidenced by the high Pliocene sedimentation rates on the continental rise (23, 24). The erosion of deep cross-shelf troughs extending to the shelf edge most likely facilitated the landward advection of warm CDW toward the WAIS’s grounding zone (60), and, thus, triggered its retreat. Glacial sea-level lowering and the associated increased incision of bathymetric cross-shelf troughs likely played one of the important roles in destabilizing the WAIS, even if atmospheric CO_2_ concentrations remained below 400 ppm and the ocean experienced only little warming. Notably, the disintegration of the Amundsen and Bellingshausen Sea drainage sectors of the WAIS at 3.46, 3.6, 3.88, 4.63 Ma proposed by this study, coincided with ice-sheet retreat in the western Ross Sea (20) and the Wilkes Subglacial Basin (21, 22) (Fig. 2*D*). Given that ice-sheet retreat in multiple sectors of West and East Antarctica could have contributed to meter-scale global sea-level rise, these periods provide crucial targets for future ice-sheet modeling coupled with ocean interactions.

Our analysis of the geochemical provenance of continental rise sediments from the Amundsen Sea revealed multiple episodes of major inland retreats of the WAIS during the Pliocene warm period, equivalent to projected future climate. The current situation, with CDW protruding to the TG–PIG grounding zones through bathymetric cross-shelf troughs, resembles the Pliocene retreat scenarios. This similarity suggests that a comparable retreat of the TG–PIG grounding lines with widespread WAIS loss may occur in the future, leading to significant sea-level rise. To further assess the stability of the WAIS, detailed records of atmospheric *p*CO_2_ and bottom water temperature on the ASE shelf are crucial for future research, especially at 3.33 Ma, 3.46 Ma, 3.6 Ma, 3.88 Ma, and 4.63 Ma when major WAIS retreat occurred.

## Materials and Methods

A full description of methods is included in the *SI Appendix*, Text File.

### Age Model.

Core contains 14 paleomagnetic reversals within the Pliocene interval (45.41 to 580.81 m), which were correlated to the GTS2012 (23, 27). Calcareous nannofossils and foraminifera are generally absent at Site U1532. Although diatom and radiolarian datums exist for seven stratigraphic sections between 4.63 Ma and 3.2 Ma (23) (*SI Appendix*, Fig. S2), biostratigraphic ages for Site U1532 are not well constrained because of discontinuous diatom and radiolarian presence. However, the available diatom and radiolarian datums do support the identification of paleomagnetic reversals. Consequently, only paleomagnetic reversals were used for the age–depth model for Site U1532 in this study.

We constructed an age–depth model for the studied Pliocene interval using the modeling routine, *Undatable* (61) (*SI Appendix*, Fig. S2 and Table S1). *Undatable* incorporates uncertainties in both the age and depth of paleomagnetic reversal datums. The model was run for 10^5^ iterations using a bootstrapping percentage of 10% and a Gaussian sediment accumulation rate (SAR) uncertainty factor of 0.15. This approach provides a conservative estimate of age–depth uncertainties by accounting for bootstrapping and uncertainty in SAR. The age–depth model created by *Undatable* is given in Dataset S1. Detrital isotope analyses of Site U1532 samples were conducted at 10 to 100 cm depth intervals. Given the linear sedimentation rates, a 10 cm interval represents approximately 600 to 1000 years.

### Ice-Sheet Modeling.

The ice-sheet model simulation is spatially and temporally downscaled across the Amundsen Sea, based on a continental model run spanning the Pliocene (50). This best-fit simulation is validated by a suite of marine and terrestrial ice-proximal geological records from across the continent. Following Halberstadt et al. (50), a matrix of climate model snapshots provides time-evolving temperature and precipitation fields to the ice-sheet model, simulating transient Antarctic ice-sheet evolution across the 3.6 Ma interval.

## Supplementary Material

Appendix 01 (PDF)

Dataset S01 (XLSX)

Dataset S02 (XLSX)

Dataset S03 (XLSX)

Dataset S04 (XLSX)

Dataset S05 (XLSX)

Dataset S06 (XLSX)

Dataset S07 (XLSX)

Dataset S08 (DOCX)

## Data Availability

Datasets S1–S7 contain the age model, NGR, diatom, and Sr-Nd-Pb isotope data (Site U1532, surface sediments, and bedrock samples) used in this study. Magnetic susceptibility, NGR, core images, and XRF scanning data can be downloaded from http://iodp.tamu.edu/LORE/, which is reported by Wellner et al. (23). All data are also available at https://doi.org/10.5281/zenodo.17376124 (62).
