# Supplementary material for: Repeated major inland retreat of Thwaites and Pine Island glaciers (West Antarctica) during the Pliocene
Source: Proc Natl Acad Sci U S A. 2025 Dec 22;123(1):e2508341122. doi: 10.1073/pnas.2508341122 (PMC12773727; doi:10.1073/pnas.2508341122)
Supplement: Supplementary file 1 — Appendix 01 (PDF) [file pnas.2508341122.sapp.pdf]

PNAS

Supporting Information for
Repeated major inland retreat of Thwaites and Pine Island glaciers (West Antarctica) during the Pliocene

Keiji Horikawa[a,1*], Masao Iwai[b], Claus-Dieter Hillenbrand[c], Christine S. Siddoway[d], Anna Ruth Halberstadt[e], Ellen A. Cowan[f], Michelle L. Penkrot[g], Karsten Gohl[h], Julia S. Wellner[i], Yoshihiro Asahara[j], Ki-Cheol Shin[k], Masahiro Noda[l], Miyu Fujimoto[l], and Expedition 379 Science Party[2]

Corresponding author: Keiji Horikawa[1]
Email: horikawa@sci.u-toyama.ac.jp

This PDF file includes:

Supporting text
SI References

Figures S1 to S11
Tables S1 to S2
Legends for Datasets S1 to S7

Other supporting materials for this manuscript include the following:

Datasets S1 to S7

Supporting Information Text

Materials and Methods

Location of Site U1532 and sediment lithology

We recovered a Pliocene sediment record by drilling with the R/V *JOIDES Resolution* Site U1532 on the Amundsen Sea continental rise (68°36.7'S, 107°31.5'W) during IODP Expedition 379 (1) (Dataset S1). The site is located on the western flank of Resolution Drift at 3962-m water depth and between the present day Antarctic Polar Front (APF) and the southern boundary of the Antarctic Circumpolar Current (sbACC) (2) (Fig. 1). The Resolution Drift lies directly to the East of a north-striking deep-sea channel, facilitating long-distance downslope transport of detrital material originating from the Amundsen Sea Embayment (ASE) shelf (3, 4). In general, dust flux plays a minimal role in sediment budgets near the Antarctic margins, since observed monthly mean dust concentration in Antarctica are very low (<1.2 µg m^{-3}) (5) compared to sediment concentrations (~1 kg m^{-3}) of typical glacial meltwater (6). Wengler et al. (2019) (7) also showed that no clear dust signal can be detected in the provenance of modern sediments in the vicinity of Site U1532, reflecting the detritus from West Antarctica dominates the sediment composition south of the APF.

Site U1532 was drilled to 794 m (meters below seafloor (mbsf)) and recovered nearly the entire record from the latest Miocene to Holocene, with an overall core recovery of 90% (*SI Appendix*, Figs. S1 and S2) (1). Drilling at Site U1532 had to be paused frequently due to approaching icebergs. As a consequence, 10 m-long cores were drilled from multiple holes (U1532A–U1532D and U1532G). Certain intervals of U1532 show low sediment recovery (U1532C 20X and 21X) or core disturbances (e.g., flow-in) (Fig. 2c and *SI Appendix*, Fig. S1).

The dominant feature of the Pliocene sediments at Site U1532 is the alternation of predominantly terrigenous laminated silty clay sediments and episodic occurrences of biosilica-bearing/rich, bioturbated mud with dispersed iceberg-rafted debris (IRD) (*SI Appendix*, Fig. S3). IRD-bearing muddy sediments contain open water diatoms like *Fragilariopsis* spp. (*F. barronii s.l., F. interfrigidaria, F. praeinterfrigidaria*) and *Dactyliosolen antarcticus*, and are also marked by higher productivity as indicated by higher diatom concentrations and elevated barium/titanium (Ba/Ti) ratios (*SI Appendix*, Fig. S4). A recent study of Pliocene interglacials in U1532 demonstrates increased diatom production as a direct consequence of iceberg melt (8). Some IRD-bearing mud intervals contain little to no identifiable diatoms, likely reflecting, at least partially, the loss of diatoms and other siliceous microfossils due to opal dissolution (1).

West Antarctic seafloor surface sediment and rock samples

In addition to IODP Expedition 379 core samples, this study also analyzed 42 modern seafloor surface sediment samples collected by the British Antarctic Survey (BAS) using various coring devices (mainly [giant] box corer (GBC/BC), but in some instances also gravity corer (GC), vibro-corer (VC), trigger corer (TC), and piston corer (PC)), which were provided by BAS (Cambridge, UK) and the British Ocean Sediment Core Research Facility (BOSCORF,

Southampton, UK) (Dataset S2). We primarily focus on the top 0–1 or 0–2 cm of these cores. Near-coastal shelf sediment samples offer a representation of the average isotopic composition of the subglacial bed under adjacent ice-sheet sectors because they consist of the glaciogenic detritus eroded and transported from the catchments of nearby glaciers. The majority of surface sediment samples used here are modern or of late Holocene age (9–14). At some sites, both surface and slightly deeper samples (4–6 cm) were measured and yielded similar isotope data. This consistency suggests that either the thorough homogenization of sediments by bioturbation (15, 16) or minimal changes in sediment sources and sedimentary processes during the (late) Holocene. Data from site PC505 (0–4 cm) on the lower continental rise exhibited unusually high Pb isotope values compared to surface sediments at adjacent sites. Since disturbance or loss of the uppermost sediments in piston cores are not uncommon, and the PC505 core barrels had been bent during the coring process, we cannot rule out that the PC505 core-top sediment sample is of pre-Holocene age. We therefore excluded its isotope data (PC505 (0–4 cm)) from the modern surface sediment database.

For bedrock samples (n = 100), we utilize material from the Polar Rock Repository (PRR) at Ohio State University. The selected samples cover a wide range of locations in West Antarctica, including the Antarctic Peninsula, Ellsworth Land, Marie Byrd Land (MBL), Ellsworth-Whitmore Mountains (EWM), Pensacola Mountains, and Coats Land (Dataset S3). As West Antarctica is largely covered by ice sheets, the underlying bed geology and its isotopic signature remain poorly known. To address this knowledge gap, we measured the isotopic compositions of a broad variety of bedrock collected from a geographically extensive area. We then calculated a representative, weighted mean isotopic composition for each region (Table S2, *SI Appendix*, Fig. S10). Additionally, these bedrock samples were analyzed for major element concentrations using a wavelength dispersive X-ray fluorescence (WDXRF) spectrometer.

Sr-Nd-Pb isotopes measurements

Bedrock samples (7–10 g) were crushed in an iron mortar (<1 cm pieces), and these rock fragments were then powdered in a planetary ball mill using agate balls (10 min, 380 rpm, Fritch P-6, Nagoya University, Japan). For U1532 and seafloor surface sediment samples, bulk sediment was weighed then wet-sieved (63 μm mesh nylon sieve) with ultrapure water. The fine fraction (<63 μm) was collected in a 2 L polypropylene beaker and settled for 2–3 days (or longer if needed) to ensure complete settling. The supernatant was then carefully aspirated. The remaining fine sediment was dried in an oven at 60 °C and homogenized into a powder using an agate mortar. The coarse fraction (>63 μm) was also collected and weighed after drying (60 °C). Approximately 1.0 g of fine-grained sediment was leached with 8 mL of diluted reductive solution (0.0016 M hydroxylamine hydrochloride, 0.12 M acetic acid, buffered to pH 4 with NaOH) for 5 min or 30 min on an orbital shaker at room temperature (~20 ºC). After leaching and centrifugation (10 min at 3000 rpm), the sediment was rinsed three times with ultrapure water. Biogenic opal was then leached in 10 ml of 1 M sodium hydroxide solution

(Ultrapur grade, Kanto Chemical) for 30 min at 85 °C in a water bath. Following centrifugation, aspirating the supernatant, and triple rinses in ultrapure water, the residue samples were dried in an oven at 60 °C and homogenized into a powder using an agate mortar. Rock and leached fine-grained sediment samples (~50 mg) were digested in concentrated acids (HF, HNO_3, and $HClO_4$; 1 mL each) on a hotplate (140 °C, >48 h) and then dried.

For rock samples, Sr and Pb were isolated by a Sr region column (0.5 mL, 50–100 μm, Eichrom Technologies Inc). Sr fraction was collected with 4 mL of 0.05M HNO_3, and the Pb fraction was collected with 5 mL of 6M HCl (17, 18). An MCI CHP20P resin column (0.13 mL, Supelco 13629-U) was attached to the bottom of the Sr resin column to reduce elution of organic materials from the Sr resin in both the Sr and Pb fractions. The Pb fraction of the U1532 and surface sediment samples was separated by an anion-exchange resin column (Dowex 1-X8, 100–200 mesh, 0.1 mL) (19). Samples were redissolved with 1mL of 0.5M HBr, and the solution was loaded onto the column after confirming complete dissolution. After loading the sample, the major elements were washed out with 2 mL of 0.5M HBr, and then 6M HCl was passed through to collect Pb. The Sr fraction of the U1532 and surface sediment samples and the Nd fraction of all samples were separated by using a cation-exchange resin column (Mitsubishi diaion CK08P, 75–150 μm) and an Ln-spec resin column (50–100 μm, Eichrom Technologies Inc). We employed established column chemistry procedures (20–22). Total procedural blanks for samples were less than ~100 pg for Sr, ~25 pg for Nd, and ~150 pg for Pb, and are negligible. All reagents used in the sample preparation are trace metal or ultrapure grade.

Sr, Nd, and Pb isotopic compositions were measured by a Thermo Scientific Neptune multi-collector inductively coupled plasma mass spectrometer (MC-ICP-MS) at the Research Institute for Humanity and Nature (RIHN), Kyoto, Japan. For Pb isotope analysis, samples and standards were diluted to a concentration of 40 ppb Pb in 2% HNO_3. NIST SRM 997 Tl standard solution was added to produce a 4 ppb Tl concentration (23). Instrumental mass bias was corrected by exponential normalization to $^{205}Tl/^{203}Tl = 2.38714$. Every four samples were bracketed with Ti-spiked 40 ppb Pb NIST SRM 981, and all data were normalized to NIST SRM 981 reported values ($^{206}Pb/^{204}Pb = 16.932$, $^{207}Pb/^{204}Pb = 15.485$, and $^{208}Pb/^{204}Pb = 36.675$) (24) based on these bracketing standards. Long-term NIST SRM 981 values analyzed over several years (n = 120) at RIHN are $^{206}Pb/^{204}Pb = 16.930 \pm 0.005$ (2σ), $^{207}Pb/^{204}Pb = 15.485 \pm 0.004$ (2σ) and $^{208}Pb/^{204}Pb = 36.677 \pm 0.011$ (2σ). External reproducibility of sample U1532C-6F-1W 43–45 cm (n = 4), prepared from the leaching step, yielded $^{206}Pb/^{204}Pb = 18.840 \pm 0.008$ (2σ), $^{207}Pb/^{204}Pb = 15.638 \pm 0.001$ (2σ) and $^{208}Pb/^{204}Pb = 38.730 \pm 0.015$ (2σ). For Sr isotope analysis, mass bias was corrected for using $^{86}Sr/^{88}Sr = 0.1194$ and an exponential law. Every six samples were bracketed with 100 ppb Sr NIST SRM 987, and all data were normalized to the NIST SRM 987 accepted value ($^{87}Sr/^{86}Sr$ of 0.71025, (25)) based on these bracketing standards. The long-term NIST SRM 987 value analyzed over several years at RIHN is $^{87}Sr/^{86}Sr = 0.71027 \pm 0.00006$ (n = 68, 2σ). For Nd isotope analysis, samples and standards were diluted to a concentration of 20 or 100 ppb Nd in 2% HNO_3. Sample solution

with low Nd concentration (~20 ppb) was introduced using a Cetac ARIDUS II desolvation system. The $^{143}Nd/^{144}Nd$ ratios were corrected for mass fractionation using $^{146}Nd/^{144}Nd$ = 0.7219 and an exponential law. Every five samples were bracketed with 100 ppb or 20 ppb Nd JNdi-1, and all data were normalized to the JNdi-1 accepted value ($^{143}Nd/^{144}Nd$ of 0.512115, (26)) based on these bracketing standards. The long-term JNdi-1 value analyzed over several years at RIHN is $^{143}Nd/^{144}Nd = 0.512056 \pm 0.000027$ (n = 80, 2σ). Nd isotopes are expressed as ε_{Nd}, which is expressed as $[(^{143}Nd/^{144}Nd)_{sample}/\ (^{143}Nd/^{144}Nd)_{CHUR}-1] \times 10^4$, where the chondritic uniform reservoir (CHUR) is 0.512638 (27). The external reproducibilities (2σ, e) of the Nd isotope measurements during each analytical session are given by repeated measurements on concentration-matched JNdi-1 standards. Where the internal error was larger than the external error of JNdi-1, a combined error (as 2 s.d.) was calculated by $\sqrt{(i)^2+(e)^2}$, where i is the internal error and e the external error. International rock reference materials, JB-1a (GSJ) and BCR-2 (USGS), were also analyzed over several years using the same digestion and chemical separation procedures, and the resulting Sr, Nd, and Pb isotopic compositions were within reported values (23, 28–39) (Dataset S4). Isotope data for sediment samples from Site U1532 are summarized in Dataset S5.

X-Ray Fluorescence (XRF) scanning

Archived core halves from Site U1532 were scanned at 2 cm resolution using the third generation Avaatech XRF core scanner at the IODP Gulf Coast Repository (College Station, Texas, USA) (40). For the vast majority of data, the total counts per second (cps) at each spot were >100,000 cps, but some spots yielded less than 100,000 cps, probably reflecting core disturbance or cracks. Those spot data were not used in the analysis. For this study, the total cps of potassium (K) and titanium (Ti) measured at 10 kV, and barium (Ba) measured at 50 kV, are used to determine the K/Ti and Ba/Ti ratios. The K/Ti ratio serves as a proxy for sediment provenance in Antarctic margin sediments (41, 42) due to the differing K/Ti ratios of felsic and mafic rocks. The Ba/Ti ratio reflects barium enrichment associated with increased flux of biogenic barite during interglacial periods (43, 44).

Iceberg-rafted debris (IRD) content

IRD content was measured on the same samples that were analyzed for Sr-Nd-Pb isotopes. The weight percent of the >250 µm fraction (45) was determined by sieve analysis following Cowan (2022) (46). Each sand sample was examined with a binocular microscope to estimate the volume of the iceberg-rafted sediment fraction (in volume percent) in order to exclude non-terrigenous coarse grains, such as biogenic components and manganese nodules.

Diatom assemblages

We quantitatively analyzed diatom assemblages in 202 samples from Site U1532. Freeze-dried subsamples (~0.1 g) were weighed into a 15-ml polypropylene centrifugation tube. To remove organic matter, 2-ml of 15% hydrogen peroxide (H_2O_2) was added, and the tubes were heated in a hot water bath until the reaction stopped. The samples were rinsed with distilled

water (DW) and left to settle overnight. Afterwards, the supernatant was removed, and this decantation process was repeated with DW (>5 times) every two hours until the rinse water became clear. A solution of 0.01N sodium diphosphate decahydrate ($Na_2P_2O_7 \cdot 10H_2O$, FW: 446.06) was used to improve dispersion of clay minerals. Aliquots (0.5 ml) of the mixed residue were diluted with a known volume of DW and then pipetted onto 18 x18 mm coverslips. The coverslips were dried on a horizontal paraffin stretcher at 40–60 °C. After drying, the coverslips were mounted onto microscope slides with Pluerax (Mountmedia, Wako Co.) and heated on a hotplate (>180 °C) to remove alcohol. Following a previous study (47), at least 400 diatom specimens per sample were counted. Observations were made using a Zeiss Axioskop Normarski differential interference contrast (DIC) microscope under 630x magnification with oil immersion. The number of fields of view, diatom frustules identified (including closed copula of *Denticulopsis*), and resting spores of the *Chaetoceros* group were recorded for each vertical traverse. Samples without diatoms (not even fragments) along at least three traverses, were classified as barren. Diatom concentrations were estimated using the following equation. Selected diatoms used in *SI Appendix*, Fig. S4 are reported in Dataset S6.

DC = Nd x (Ac / (Nf x Af)) x (dW/dR) [#/g-dry sediment]

where,

DC: diatom concentration (#/g-dry sediment)
Nd: number of diatoms counted
Nf: number of fields observed
Ac: area of cover slip (18 x 18 mm^2)
Af: area of a field view (mm^2)
dW: dry-weight of subsample sediment used for processing (g-dry sediment)
dR: dilution ratio (0.5/10 when spread 0.5-ml from 10-ml mixed residues)

SI References

1. J. S. Wellner *et al.*, "Site U1532" in *Proceedings of the International Ocean Discovery Program Volume 379*, K. Gohl, J. S. Wellner, A. Klaus, the Expedition 379 Scientists, Eds. (International Ocean Discovery Program, 2021).
2. A. H. Orsi, T. Whitworth, W. D. Nowlin, On the meridional extent and fronts of the Antarctic Circumpolar Current. *Deep-Sea Res. I* **42**, 641–673 (1995).
3. K. Gohl *et al.*, Evidence for a highly dynamic West Antarctic Ice Sheet during the Pliocene. *Geophys. Res. Lett.* **48**, e2021GL093103 (2021).
4. J. A. Dowdeswell, J. Evans, C. Ó Cofaigh, J. B. Anderson, Morphology and sedimentary processes on the continental slope off Pine Island Bay, Amundsen Sea, West Antarctica. *GSA Bulletin* **118**, 606–619 (2006).
5. F. Li, P. Ginoux, V. Ramaswamy, Distribution, transport, and deposition of mineral dust in the Southern Ocean and Antarctica: Contribution of major sources. *J. Geophys. Res. Atmospheres* **113**, 2007JD009190 (2008).
6. R. Raiswell *et al.*, Contributions from glacially derived sediment to the global iron (oxyhydr)oxide cycle: Implications for iron delivery to the oceans. *Geochim. Cosmochim. Acta.* **70**, 2765–2780 (2006).
7. M. Wengler *et al.*, A geochemical approach to reconstruct modern dust fluxes and sources to the South Pacific. *Geochim. Cosmochim. Acta.* **264**, 205–223 (2019).
8. H. Furlong, R. P. Scherer, Direct link between iceberg melt and diatom productivity demonstrated in Mid-Pliocene Amundsen Sea interglacial sediments. *J. Micropalaeontol.* **43**, 269–282 (2024).
9. P. Simões Pereira *et al.*, Geochemical fingerprints of glacially eroded bedrock from West Antarctica: Detrital thermochronology, radiogenic isotope systematics and trace element geochemistry in Late Holocene glacial-marine sediments. *Earth-Sci. Rev.* **182**, 204–232 (2018).
10. J. A. Smith *et al.*, New constraints on the timing of West Antarctic Ice Sheet retreat in the eastern Amundsen Sea since the Last Glacial Maximum. *Global Planet. Change* **122**, 224–237 (2014).
11. R. D. Larter *et al.*, Reconstruction of changes in the Amundsen Sea and Bellingshausen Sea sector of the West Antarctic Ice Sheet since the Last Glacial Maximum. *Quat. Sci. Rev.* **100**, 55–86 (2014).
12. J. A. Smith *et al.*, Deglacial history of the West Antarctic Ice Sheet in the western Amundsen Sea Embayment. *Quat. Sci. Rev.* **30**, 488–505 (2011).

13. C.-D. Hillenbrand *et al.*, The sedimentary legacy of a palaeo-ice stream on the shelf of the southern Bellingshausen Sea: Clues to West Antarctic glacial history during the Late Quaternary. *Quat. Sci. Rev.* **29**, 2741–2763 (2010).

14. C.-D. Hillenbrand *et al.*, Grounding-line retreat of the West Antarctic Ice Sheet from inner Pine Island Bay. *Geology* **41**, 35–38 (2013).

15. J. A. Smith *et al.*, The marine geological imprint of Antarctic ice shelves. *Nat. Commun.* **10**, 5635 (2019).

16. A. M. Dolman, J. Groeneveld, G. Mollenhauer, S. L. Ho, T. Laepple, Estimating bioturbation from replicated small-sample radiocarbon ages. *Paleoceanogr. Paleoclimatol.* **36**, e2020PA004142 (2021).

17. Wakaki, S., Kawai, T., Nagaishi, K., Ishikawa, T., Sequential chemical separation of Sr, Nd and Pb from geological samples using multi-step extraction column chromatography. *JAMSTEC-R* **27**, 1–12 (2018).

18. A. Makishima, B. N. Nath, E. Nakamura, New sequential separation procedure for Sr, Nd and Pb isotope ratio measurement in geological material using MC-ICP-MS and TIMS. *Geochem. J.* **42**, 237–246 (2008).

19. M. M. De Mahiques *et al.*, Nd and Pb isotope signatures on the Southeastern South American upper margin: Implications for sediment transport and source rocks. *Mar. Geol.* **250**, 51–63 (2008).

20. K. Yamamoto, F. Yamashita, M. Adachi, Precise determination of REE for sedimentary reference rocks issued by the Geological Survey of Japan. *Geochem. J.* **39**, 289–297 (2005).

21. K. Horikawa *et al.*, Neodymium isotope records from the Northwestern Pacific: Implication for deepwater ventilation at Heinrich Stadial 1. *Paleoceanogr. Paleoclimatol.* **36**, 2021PA004312 (2021).

22. C. Pin, J. S. Zalduegui, Sequential separation of light rare-earth elements, thorium and uranium by miniaturized extraction chromatography: Application to isotopic analyses of silicate rocks. *Anal. Chim. Acta* **339**, 79–89 (1997).

23. M. Tanimizu, T. Ishikawa, Development of rapid and precise Pb isotope analytical techniques using MC-ICP-MS and new results for GSJ rock reference samples. *Geochem. J.* **40**, 121–133 (2006).

24. A. Cocherie, M. Robert, Direct measurement of lead isotope ratios in low concentration environmental samples by MC-ICP-MS and multi-ion counting. *Chem. Geol.* **243**, 90–104 (2007).

25. G. Faure, T. M. Mensing, *Principles and applications* (John Wiley & Sons, Inc, 2005).

26. T. Tanaka *et al.*, JNdi-1: a neodymium isotopic reference in consistency with LaJolla neodymium. *Chem. Geol.* **168**, 279–281 (2000).

27. S. B. Jacobsen, G. J. Wasserburg, Sm-Nd isotopic evolution of chondrites. *Earth Planet. Sci. Lett.* **50**, 139–155 (1980).

28. J. Jweda, L. Bolge, C. Class, S. L. Goldstein, High precision Sr-Nd-Hf-Pb isotopic compositions of USGS reference material BCR-2. *Geostand. Geoanal. Res.* **40**, 101–115 (2016).

29. J. D. Woodhead, J. M. Hergt, Pb-Isotope analyses of USGS reference materials. *Geostand. Geoanal. Res.* **24**, 33–38 (2000).

30. Y. Orihashi, J. Maeda, R. Tanaka, R. Zeniya, K. Niida, Sr and Nd isotopic data for the seven GSJ rock reference samples; JA-1, JB-1a, JB-2, JB-3, JG-1a, JGb-1 and JR-1. *Geochem. J.* 205–211 (1998).

31. D. Weis *et al.*, High-precision isotopic characterization of USGS reference materials by TIMS and MC-ICP-MS. *Geochem. Geophys. Geosyst.* **7**, 2006GC001283 (2006).

32. Y. V. Erban Kochergina, V. Erban, J. M. Hora, Sample preparation and chromatographic separation for Sr, Nd, and Pb isotope analysis in geological, environmental, and archaeological samples. *Jour. Geosci.* **67**, 273–285 (2022).

33. K. Guo *et al.*, Precise determination of Sr and Nd isotopic compositions of Chinese Standard Reference samples GSR-1, GSR-2, GSR-3 and GBW07315 by TIMS. *Geosystems and Geoenvironment* **2**, 100206 (2023).

34. A. M. Jewell *et al.*, Three North African dust source areas and their geochemical fingerprint. *Earth Planet. Sci. Lett.* **554**, 116645 (2021).

35. C.-F. Li, X.-C. Wang, J.-H. Guo, Z.-Y. Chu, L.-J. Feng, Rapid separation scheme of Sr, Nd, Pb, and Hf from a single rock digest using a tandem chromatography column prior to isotope ratio measurements by mass spectrometry. *J. Anal. At. Spectrom.* **31**, 1150–1159 (2016).

36. C. Li, Z. Chu, X. Wang, J. Guo, S. A. Wilde, Determination of $^{87}Rb/^{86}Sr$ and $^{87}Sr/^{86}Sr$ ratios and Rb–Sr contents on the same filament loading for geological samples by isotope dilution thermal ionization mass spectrometry. *Talanta* **233**, 122537 (2021).

37. M. Yamamoto *et al.*, Sr and Nd isotopic compositions of mafic xenoliths and volcanic rocks from the Oga Peninsula, Northeast Japan Arc: Genetic relationship between lower crust and arc magmas. *Lithos* **162–163**, 88–106 (2013).

38. R. Fukai, T. Yokoyama, Nucleosynthetic Sr–Nd Isotope correlations in chondrites: evidence for nebular thermal processing and dust transportation in the early solar system. *ApJ* **879**, 79 (2019).

39. K. P. Jochum, U. Nohl, Reference materials in geochemistry and environmental research and the GeoReM database. *Chem. Geol.* **253**, 50–53 (2008).

40. M. Lyle, D. K. Kulhanek, M. G. Bowen, A. Hahn, "Data report: X-ray fluorescence studies of Site U1457 sediments, Laxmi Basin, Arabian Sea" in *Proceedings of the International Ocean Discovery Program Volume 355*, D. K. Pandey, P. D. Clift, D. K. Kulhanek, the Expedition 355 Scientists, Eds. (International Ocean Discovery Program, 2018).

41. D. Monien, G. Kuhn, H. von Eynatten, F. M. Talarico, Geochemical provenance analysis of fine-grained sediment revealing Late Miocene to recent Paleo-Environmental changes in the Western Ross Sea, Antarctica. *Global Planet. Change* **96–97**, 41–58 (2012).

42. R. A. Bertram *et al.*, Pliocene deglacial event timelines and the biogeochemical response offshore Wilkes Subglacial Basin, East Antarctica. *Earth Planet. Sci. Lett.* **494**, 109–116 (2018).

43. W. J. Bonn, F. X. Gingele, H. Grobe, A. Mackensen, D. K. Fütterer, Palaeoproductivity at the Antarctic continental margin: opal and barium records for the last 400 ka. *Palaeogeogr. Palaeoclimatol. Palaeoecol.* **139**, 195–211 (1998).

44. C.-D. Hillenbrand, G. Kuhn, T. Frederichs, Record of a Mid-Pleistocene depositional anomaly in West Antarctic continental margin sediments: An indicator for ice-sheet collapse? *Quat. Sci. Rev.* **28**, 1147–1159 (2009).

45. R. McKay *et al.*, A comparison of methods for identifying and quantifying ice rafted debris on the Antarctic margin. *Paleoceanogr. Paleoclimatol.* **37**, e2021PA004404 (2022).

46. E. A. Cowan, Unraveling the deep-sea sedimentary record of ice sheet history. *Paleoceanogr. Paleoclimatol.* **37**, e2022PA004488 (2022).

47. H. Schrader, R. Gersonde, Diatoms and silicoflagellates. *In Zachariasse etb al. Microplaeontological counting methods and techniques-an excercise on an eight metres section of the lower Pliocene of Capo Rossello. Sicily. Utrecht Micropal. Bull* **17**, 129–176 (1978).

48. T. Westerhold *et al.*, An astronomically dated record of Earth's climate and its predictability over the last 66 million years. *Science* **369**, 1383–1387 (2020).

49. J. G. Ogg, "Chapter 5 - Geomagnetic Polarity Time Scale" in *The Geologic Time Scale*, F. M. Gradstein, J. G. Ogg, M. D. Schmitz, G. M. Ogg, Eds. (Elsevier, 2012), pp. 85–113.

50. B. C. Lougheed, S. P. Obrochta, A rapid, deterministic age-depth modeling routine for geological wequences with inherent depth uncertainty. *Paleoceanogr. Paleoclimatol.* **34**, 122–133 (2019).

51. P. Simões Pereira *et al.*, The geochemical and mineralogical fingerprint of West Antarctica's weak underbelly: Pine Island and Thwaites glaciers. *Chem. Geol.* **550**, 119649 (2020).

52. A. E. Carlson, B. L. Beard, R. G. Hatfield, M. Laffin, Absence of West Antarctic-sourced silt at ODP Site 1096 in the Bellingshausen Sea during the last interglaciation: Support for West Antarctic ice-sheet deglaciation. *Quat. Sci. Rev.* **261**, 106939 (2021).

53. G. L. Farmer, K. Licht, R. Swope, J. Andrews, Isotopic constraints on the provenance of fine-grained sediment in LGM tills from the Ross Embayment, Antarctica. *Earth Planet. Sci. Lett.* **249**, 90–107 (2006).

54. R. P. Caballero-Gill, T. D. Herbert, H. J. Dowsett, 100-kyr paced climate change in the Pliocene warm period, Southwest Pacific. *Paleoceanogr. Paleoclimatol.* **34**, 524–545 (2019).

55. S. Hou *et al.*, Lipid-biomarker-based sea surface temperature record offshore Tasmania over the last 23 million years. *Clim. Past* **19**, 787–802 (2023).

56. J. Laskar *et al.*, A long-term numerical solution for the insolation quantities of the Earth. *A&A* **428**, 261–285 (2004).

57. K. Matsuoka *et al.*, Quantarctica, an integrated mapping environment for Antarctica, the Southern Ocean, and sub-Antarctic islands. *Environ. Model. Softw.* **140**, 105015 (2021).

58. R. J. Tingey, 1:10 million scale Continent-wide surface schematic geological units and ages, compiled in 1985-1986. (1991).

59. S. R. Hart, A large-scale isotope anomaly in the Southern Hemisphere mantle. *Nature* **309**, 753–757 (1984).

60. J. S. Stacey, J. D. Kramers, Approximation of terrestrial lead isotope evolution by a two-stage model. *Earth Planet. Sci. Lett.* **26**, 207–221 (1975).

61. J. W. Marschalek *et al.*, Byrd Ice Core Debris Constrains the Sediment Provenance Signature of Central West Antarctica. *Geophys. Res. Lett.* **51**, e2023GL106958 (2024).

62. Y. Kato, I. Hernández-Almeida, L. F. Pérez, Diatom and radiolarian biostratigraphy in the Pliocene sequence of ODP Site 697 (Jane Basin, Atlantic sector of the Southern Ocean). *J. Micropalaeontol.* **43**, 93–119 (2024).

Supporting Information Figures

Fig. S1

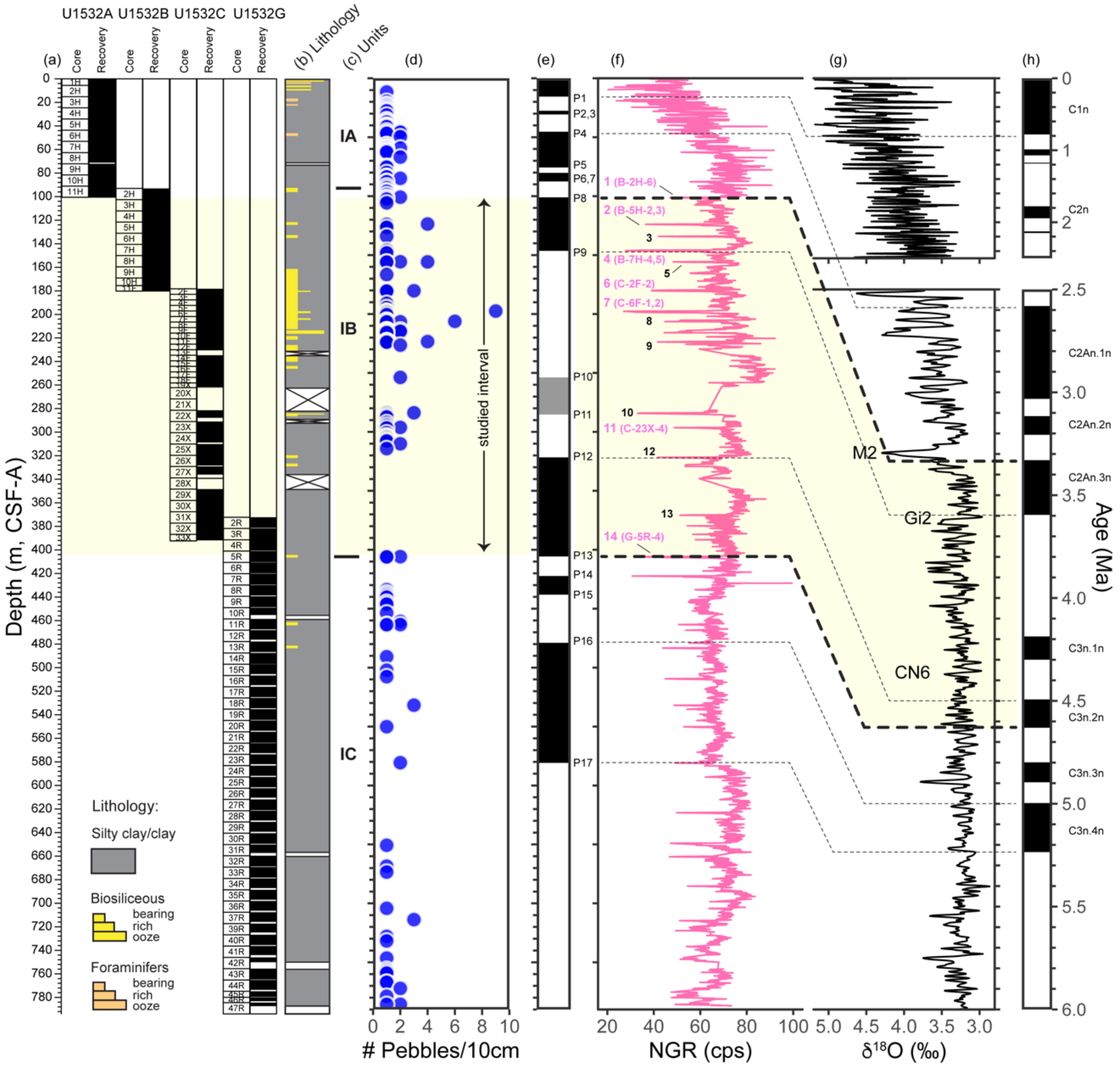

Composite lithostratigraphic summary of Site U1532. (a) Core recovery at each Hole of Site U1532. (b, c) Schematic lithology of cores from Site U1532. Major lithologies are divided into Subunits IA, IB, and IC based on changes in facies assemblages (1). (d) Number of pebbles within each 10 cm interval. (e) Paleomagnetic polarity of cores from Site U1532. P1–P17 are the paleomagnetic reversals identified onboard (1). Paleomagnetic measurements for Hole U1532C identified the termination of the Nunivak Subchron (C3n.2n, 4.493 Ma) but no clear Cochiti Subchron (C3n.1n, 4.187–4.300 Ma) due to limited core recovery in this interval (~256–290 m). However, inclination values obtained from discrete sample analysis in this interval suggest continuous normal polarity. Therefore, the paleomagnetic reversals P10 and P11 were also used for the age model of the studied interval. (f) Natural gamma ray (NGR) record from Site U1532 (Dataset S7). Lower NGR values indicate IRD-bearing mud intervals deposited during interglacials marked by major WAIS retreat. (g) Global deep-sea benthic foraminiferal $\delta^{18}O$ stack (48) and labels for some glacial stages. (h) Paleomagnetic data (black = normal, white = reversed)

(49). Not all correlation lines between the magnetic polarity zones of Site U1532 and the GTS2012 are shown.

Fig. S2

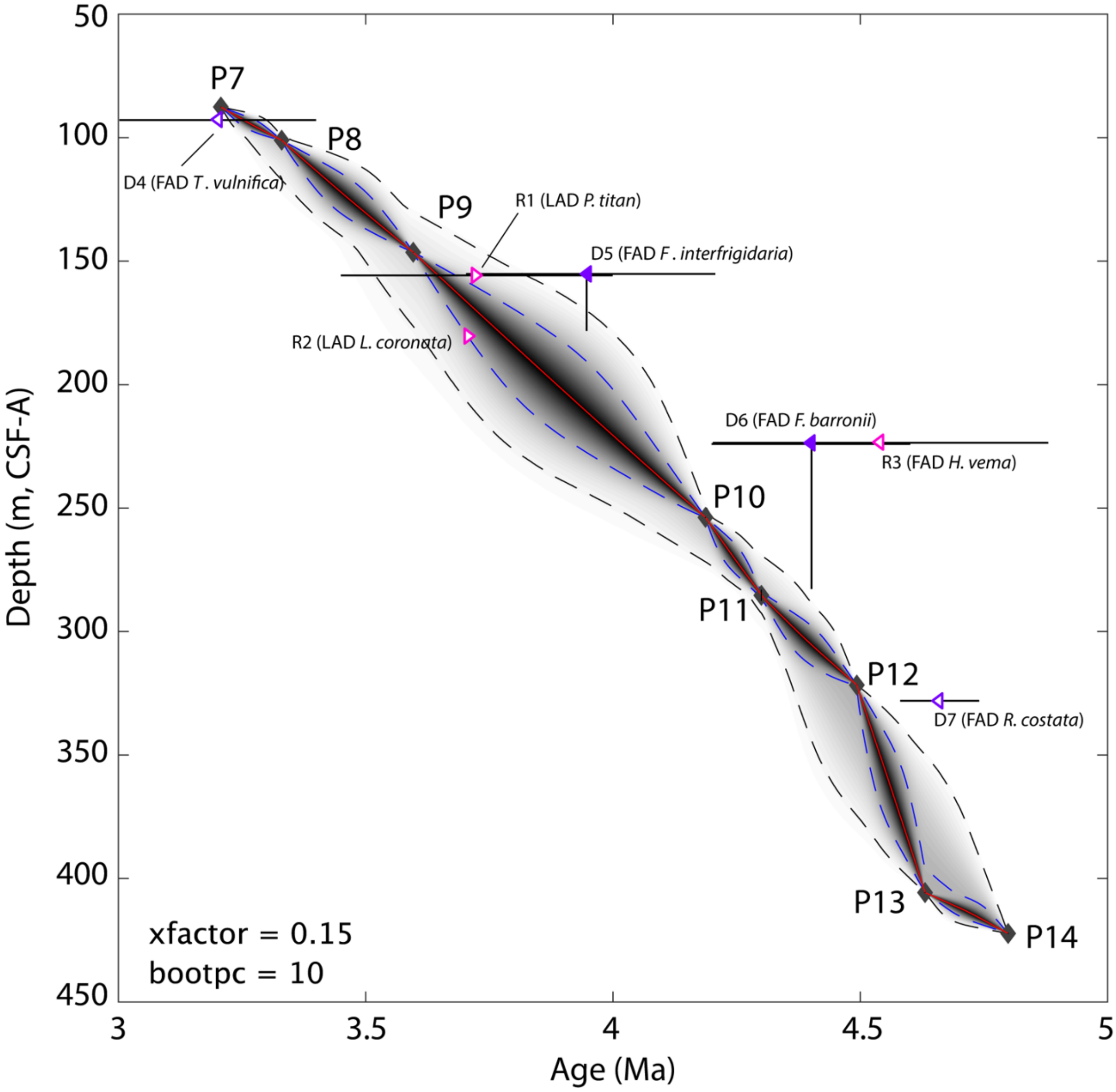

Age-depth model for the Pliocene section of Site U1532. Age-depth model for the Pliocene section of Site U1532 with bootstrapping set to 10% and sedimentation rate uncertainty set to 0.15 (see (50) for details). Diamonds indicate the tie points of paleomagnetic reversals (P7–P14 in (1)). The gray cloud indicates the probability density cloud of the age-depth model, whereby darker colors indicate higher age-depth probability. The blue and black broken lines represent 68.2% and 95.4% confidence intervals, respectively. The red line indicates the age-depth model median. Diatom and radiolarian biostratigraphic datums identified at Site U1532 are also indicated (1). LAD = last appearance datum, FAD = first appearance datum.

Fig. S3

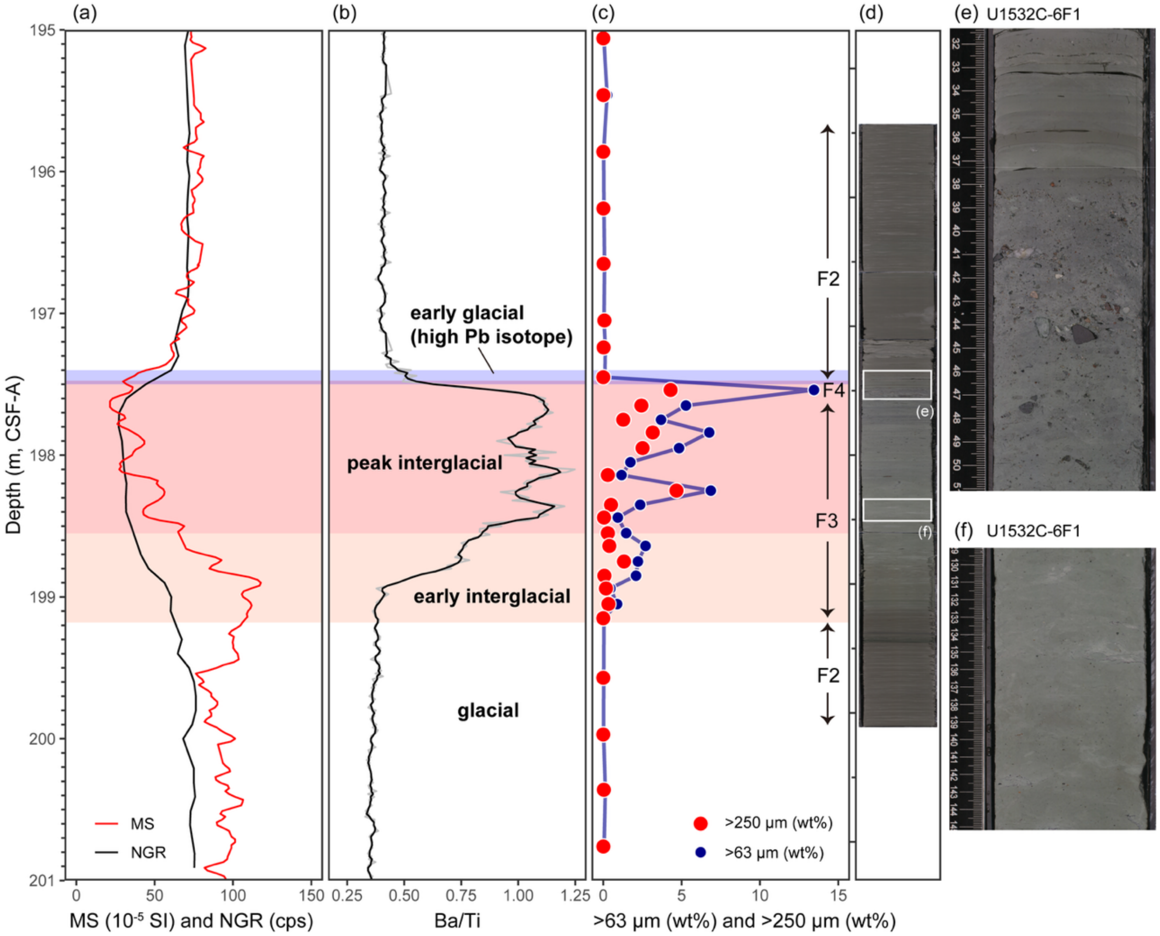

Examples of lithological features associated with melt events during a glacial–interglacial cycle. F2, F3, F4 lithofacies from (1). The Pliocene sediments are marked by alternations of thick, gray, predominantly terrigenous laminated silty clays (F2) with relatively thin, greenish, biosilica-bearing/rich, bioturbated muds containing dispersed iceberg-rafted debris (IRD) (F3 and F4 in (1)) (d). These IRD-bearing greenish mud intervals are characterized by lower NGR and magnetic susceptibility (MS) values (a) and negative a*-values (1) (Fig. 2a). IRD-containing F3 and F4 intervals also exhibit higher productivity, as indicated by higher diatom concentrations (*SI Appendix*, Fig. S4) and elevated Ba/Ti ratios (b). IRD content usually increases towards the top of the greenish muds (c, e, f).

Fig. S4

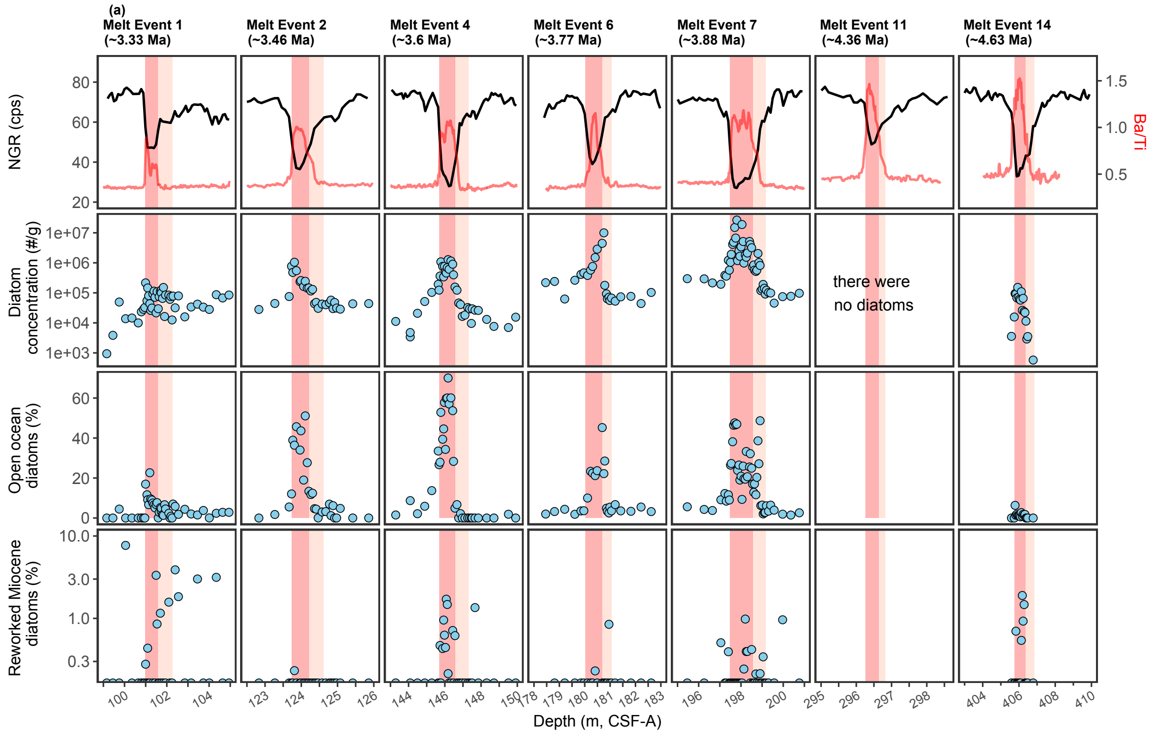

Records of NGR, Ba/Ti ratios, and diatom content across seven of the prominent Pliocene interglacial periods at Site U1532. Very light red and light red shading highlight early and peak interglacial stages, respectively, which are marked by IRD-bearing mud intervals. NGR data and Ba/Ti ratios are shown by black and red lines, respectively. Element ratios represent 3-point running means of 2 cm interval data points. Open seawater diatom taxa comprise *Fragilariopsis* spp. (*F. barronii s.l., F. interfrigidaria, F. praeinterfrigidaria*) and *Dactyliosolen antarcticus*. Reworked Miocene diatom taxa (*Nitzschia denticuloide* and *Nitzschia grossepunctata*) indicate reworking from middle Miocene sediments. Diatom assemblage data is unavailable for the 4.36 Ma interval due to poor diatom preservation.

Fig. S5

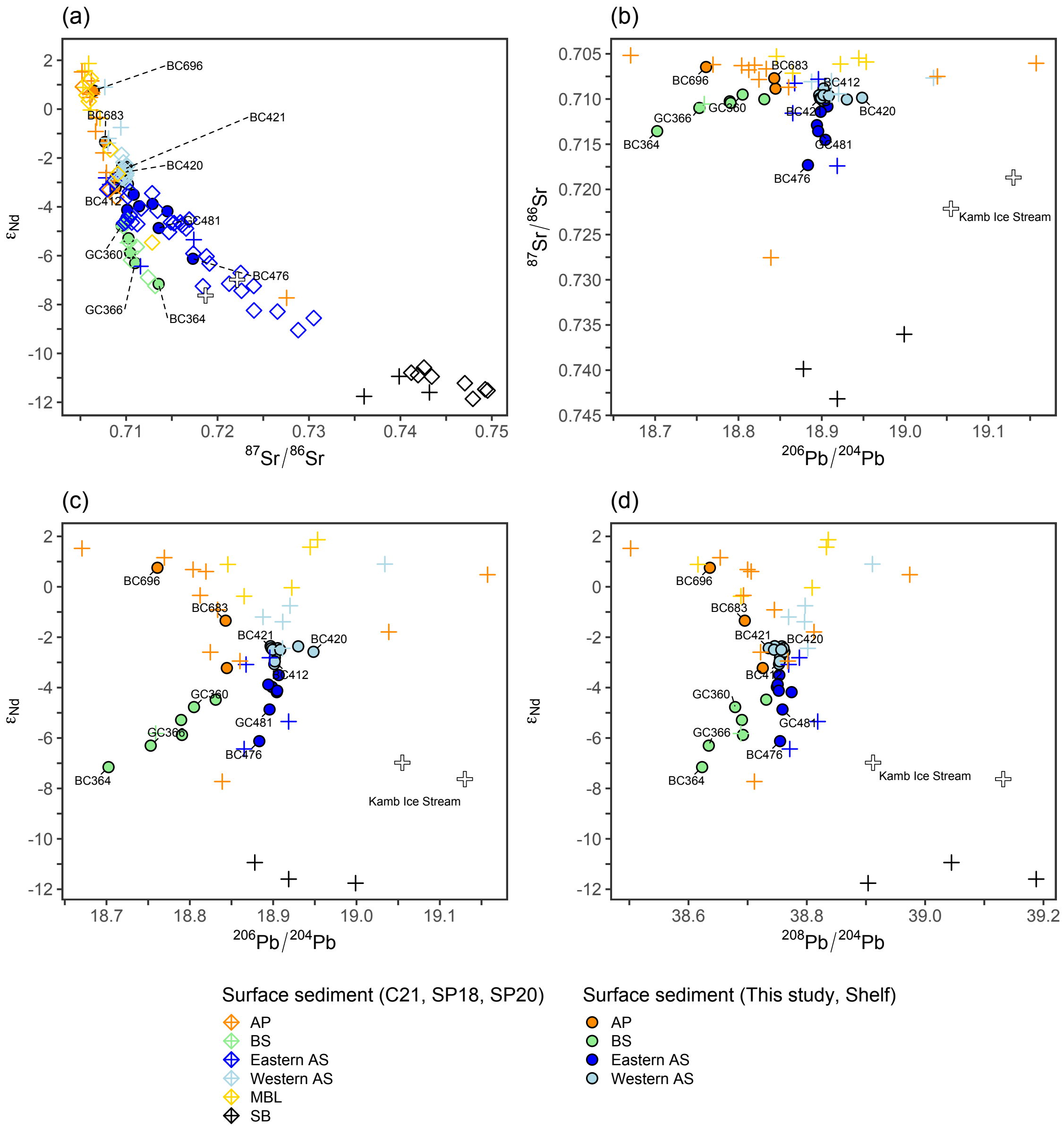

Provenance of modern seafloor surface sediments on the West Antarctic margin. Isotopic compositions of modern seafloor surface sediments at shelf sites near the western Antarctic Peninsula (AP), in the Bellingshausen Sea (BS) and the eastern and western Amundsen Sea (AS), including seafloor surface sample data from Simões Pereira et al. (2018) and Simões Pereira et al. (2020) (SP18 and SP20, diamond (9, 51)), Carlson et al. (2021) (C21, cross (52)), and subglacial data from Farmer et al. (2006) (open cross (53)). (a) ε_{Nd} versus $^{87}Sr/^{86}Sr$, (b) $^{87}Sr/^{86}Sr$ versus $^{206}Pb/^{204}Pb$, (c) ε_{Nd} versus $^{206}Pb/^{204}Pb$, and (d) ε_{Nd} versus $^{208}Pb/^{204}Pb$. MBL and SB are Marie Byrd Land and Sulzberger Bay, respectively. Selected ice-sheet proximal sites are labeled with their IDs (Fig. 1).

Fig. S6

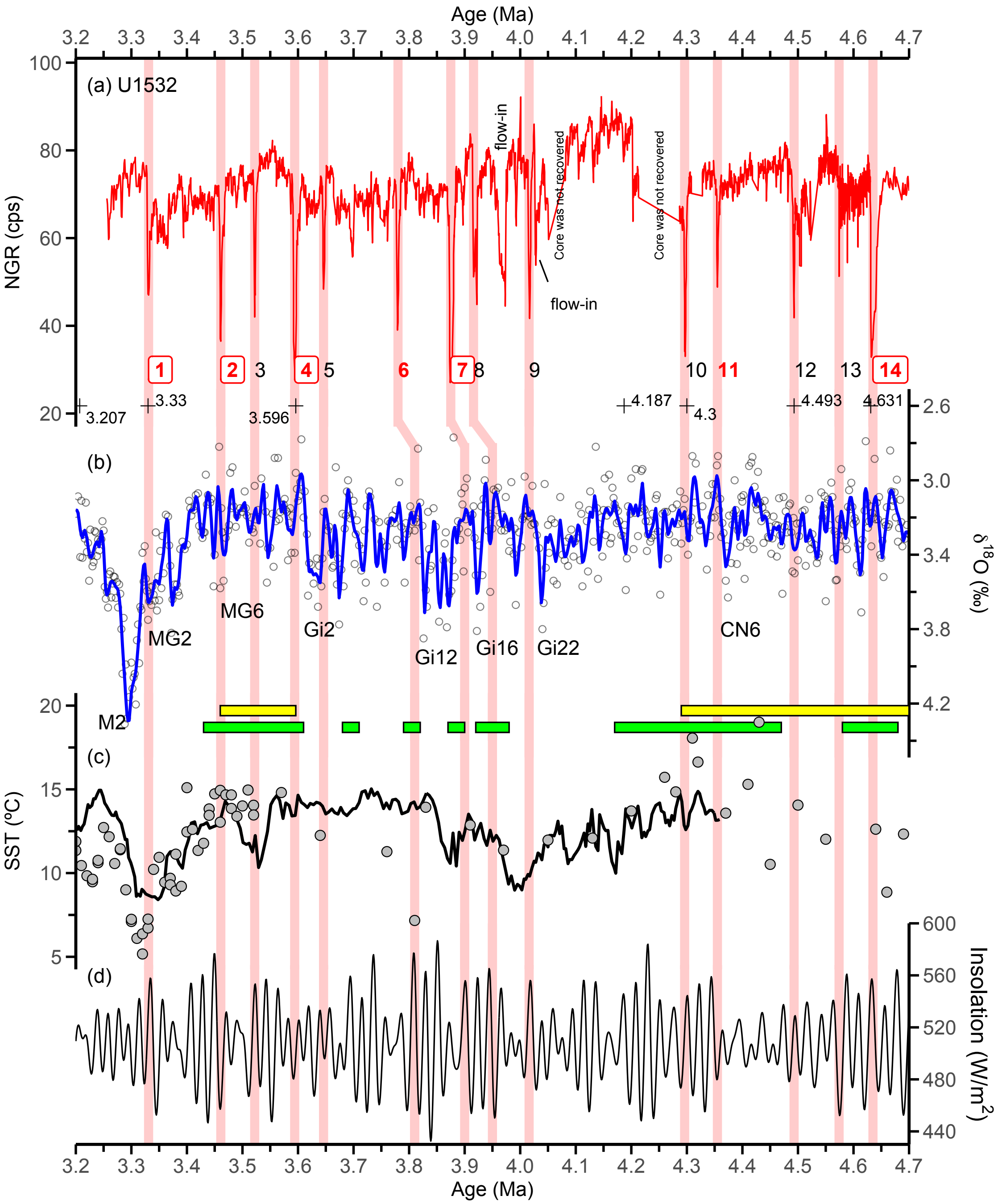

Records of NGR at Site U1532, benthic foraminiferal δ^{18}O stack, sea-surface temperature, and insolation between 4.7 and 3.2 Ma. (a) NGR record from Site U1532. Lower NGR values indicate IRD-bearing mud intervals deposited during interglacials marked by major WAIS retreat. Fourteen melt events (numbered) were identified between 4.7 Ma and 3.2 Ma. Numbers enclosed in squares represent major inland retreat events of the WAIS. Provenance was analyzed by measuring isotope characteristics of fine-grained detritus from seven intervals (1, 2, 4, 6, 7, 11, and 14, shown in red font) (Fig. 2). (b) Global deep-sea benthic foraminiferal δ^{18}O stack (48). (c) Sea-surface temperature (SST) at Deep-Sea Drilling

Project (DSDP) Site 594 (45º31.41'S, 174º56.88'E, black line (54) and Ocean Drilling Program (ODP) Site 1168 (42º36.58'S, 144º24.76'E, gray circles (55)). (d) Mean monthly insolation at 80ºS (21 December–20 January) (56).

Fig. S7

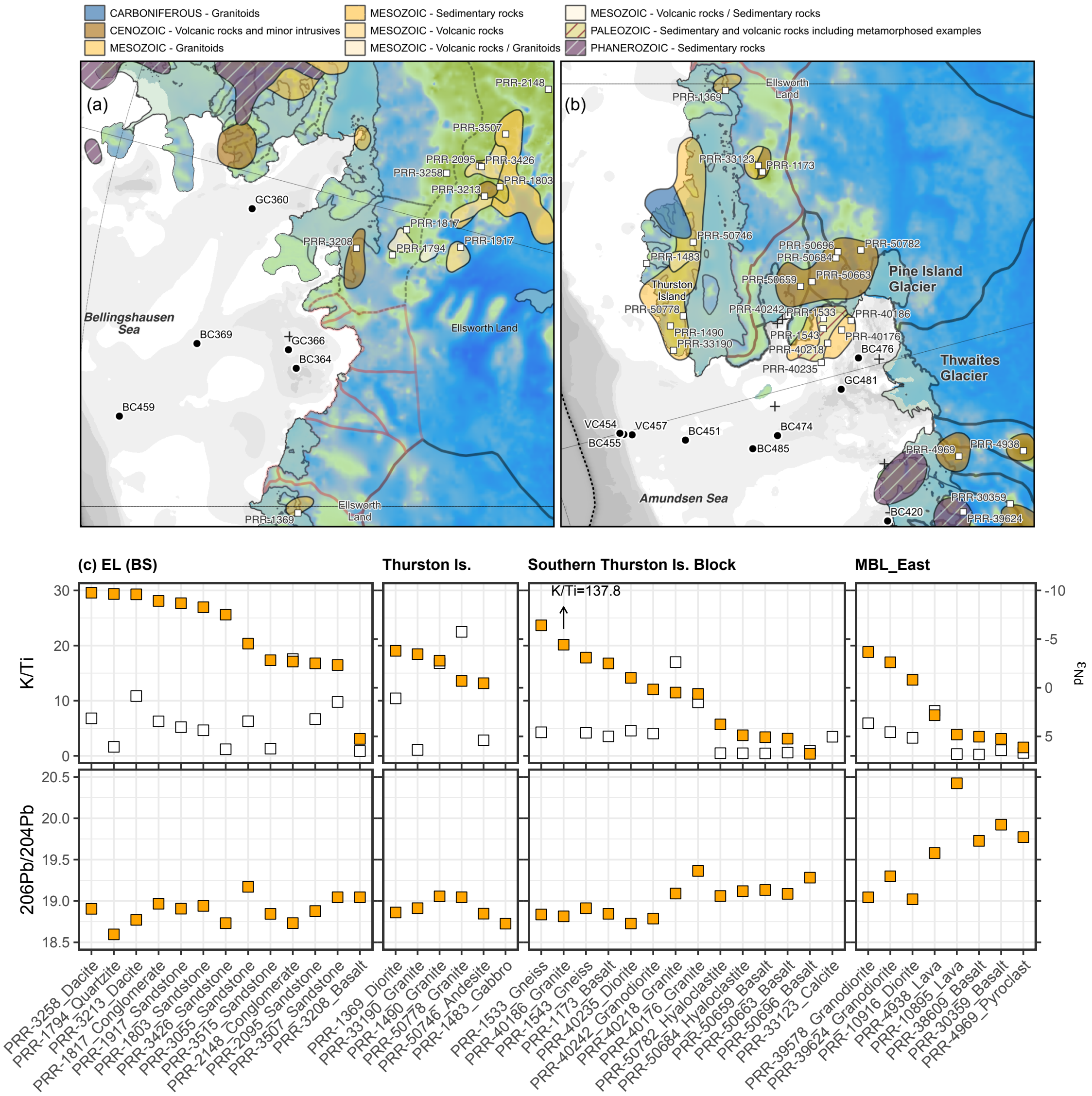

Locations and geochemical characteristics of bedrock samples from the Bellingshausen and Amundsen Sea regions. Maps of (a) the Bellingshausen Sea and (b) Amundsen Sea areas. Locations are shown for bedrock samples (white squares) and seafloor surface sediment samples (black circles). Bedrock samples, some collected from islands, are labeled with their Polar Rock Repository identifier (PRR #). (c) Geochemical data for individual bedrock samples from four geographic source regions, showing rock types, K/Ti (open squares), ε_{Nd} values (orange squares, upper panels), and $^{206}Pb/^{204}Pb$ isotope ratios (orange squares, lower panels). The map was created by QGIS (57), and schematic geological map is from Bureau of Mineral Resources, Geology and Geophysics (1991) (58). Weighted mean and standard deviation values for each region are given in Table S2. See *SI Appendix* Fig. S10 for all isotope data in the eleven geographic areas.

Fig. S8

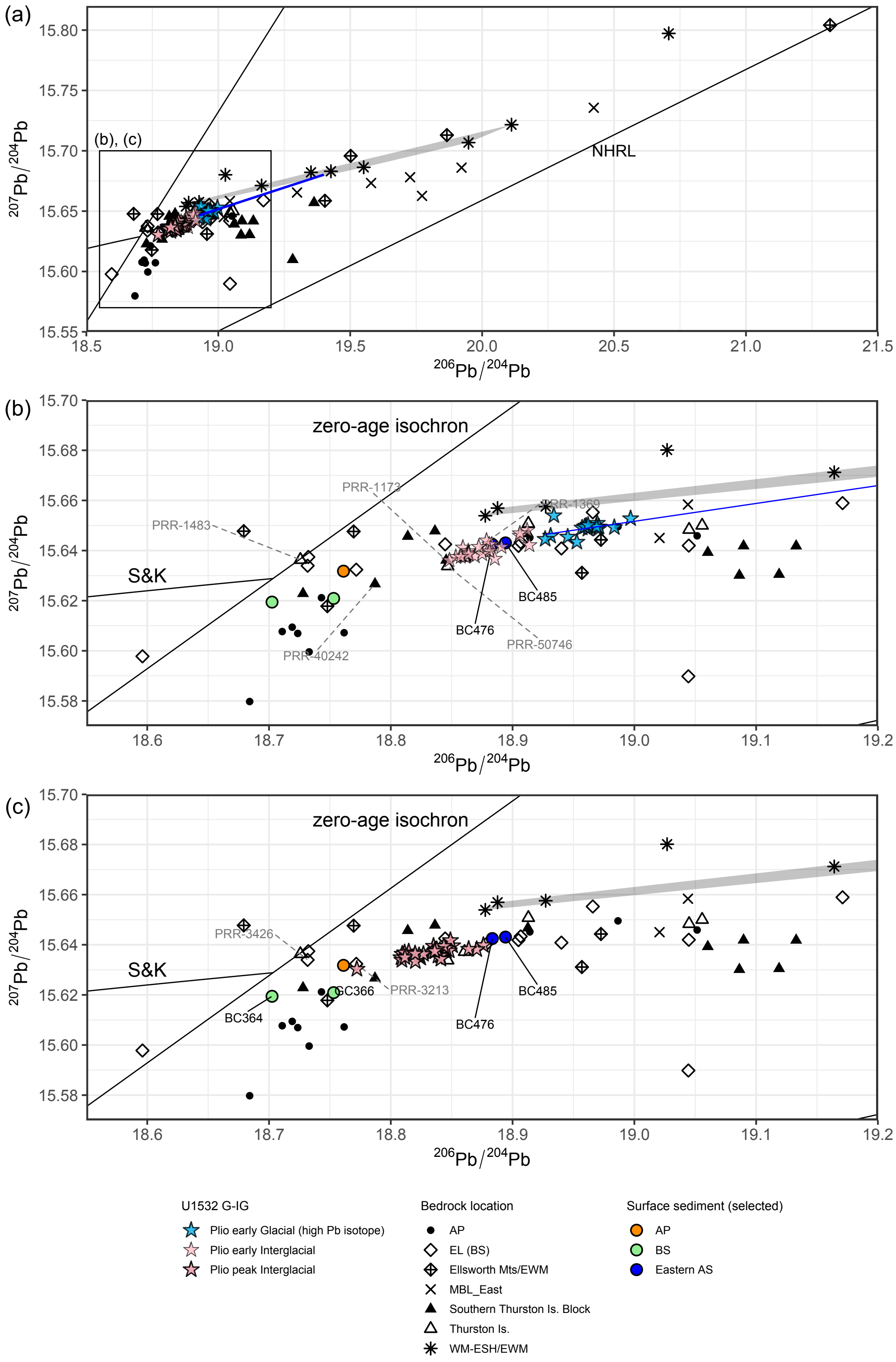

(a)
^{207}Pb/^{204}Pb
^{206}Pb/^{204}Pb
(b), (c)
NHRL
(b)
zero-age isochron
S&K
PRR-1483
PRR-1173
PRR-1369
PRR-40242
PRR-50746
BC485
BC476
(c)
PRR-3426
PRR-3213
BC364
BC366
U1532 G-IG
Plio early Glacial (high Pb isotope)
Plio early Interglacial
Plio peak Interglacial
Bedrock location
AP
EL (BS)
Ellsworth Mts/EWM
MBL_East
Southern Thurston Is. Block
Thurston Is.
WM-ESH/EWM
Surface sediment (selected)
AP
BS
Eastern AS

$^{206}Pb/^{204}Pb$ vs $^{207}Pb/^{204}Pb$ systematics for Site U1532 sediments and regional bedrock. Reference lines include Northern Hemisphere Reference Line (NHRL(59)) and the two-stage terrestrial Pb evolution model (S&K(60)). The gray shaded field represents the granites from the Whitmore Mountains–Ellsworth Subglacial Highlands in the Ellsworth-Whitmore Mountains (WM-ESH/EWM). Bedrock data are shown for key geographic regions (AP: Antarctic Peninsula; BS: Bellingshausen Sea; EL: Ellsworth Land; MBL: Marie Byrd Land). Data from surface sediment samples from several key shelf sites are also shown, with core IDs labelled in panels (b) and (c). (a) Data for Pliocene early glacial sediments (characterized by high Pb isotope ratios), early interglacial sediments, and peak interglacial sediments are shown. (b) Detailed view of Pb-isotope data from early glacial sediments and early interglacial sediments. The early glacial data form a linear array (blue regression line) that intersects the WM-ESH/EWM granite field at a $^{206}Pb/^{204}Pb$ value of ~19.4. This supports the interpretation that the WM-ESH/EWM granites, acting as the radiogenic endmember, were a greater source component in the early glacial samples (see also Fig. S11). Bedrock samples from eastern Marie Byrd Land (MBL_East), which are also characterized by high Pb-isotope ratios, possess ε_{Nd} values (–3.6 to +6.1 ε_{Nd}) that are too high to produce the low ε_{Nd} values observed in the early glacial sediments (~–6 ε_{Nd}) at Site U1532. In contrast, the early interglacial data scatter around those from samples BC476 and BC485 and tend towards Pb-isotope ratios lower than the early glacial samples but higher than the peak interglacial samples (c), plotting between bedrock data fields from the Southern Thurston Island block (closed triangles) and Thurston Island (open triangles). The shift toward less radiogenic values suggests an increased contribution from less radiogenic sources like those represented by samples PRR-50746, -1369, -1483, -40242, and -1173. (c) Detailed view of Pb-isotope data from peak interglacial sediments. The peak interglacial data appear to reflect an increased contribution from aforementioned sources and possibly from the coastal Bellingshausen Sea (BC364 and GC366). Additionally, some rock types represented by PRR-3426 and -3213 from the EL (BS) sector, which exhibit less radiogenic Pb isotopic ratios (18.73 for $^{206}Pb/^{204}Pb$) and low ε_{Nd} values (–9.6 to –7.5) (Fig. S7), may also have contributed to the peak interglacial sediments. Integrating the multi-proxy data (ε_{Nd}, $^{206}Pb/^{204}Pb$, and K/Ti ratios) presented in Figures 3b and S7 provides a more robust interpretation of these complex sources.

Fig. S9

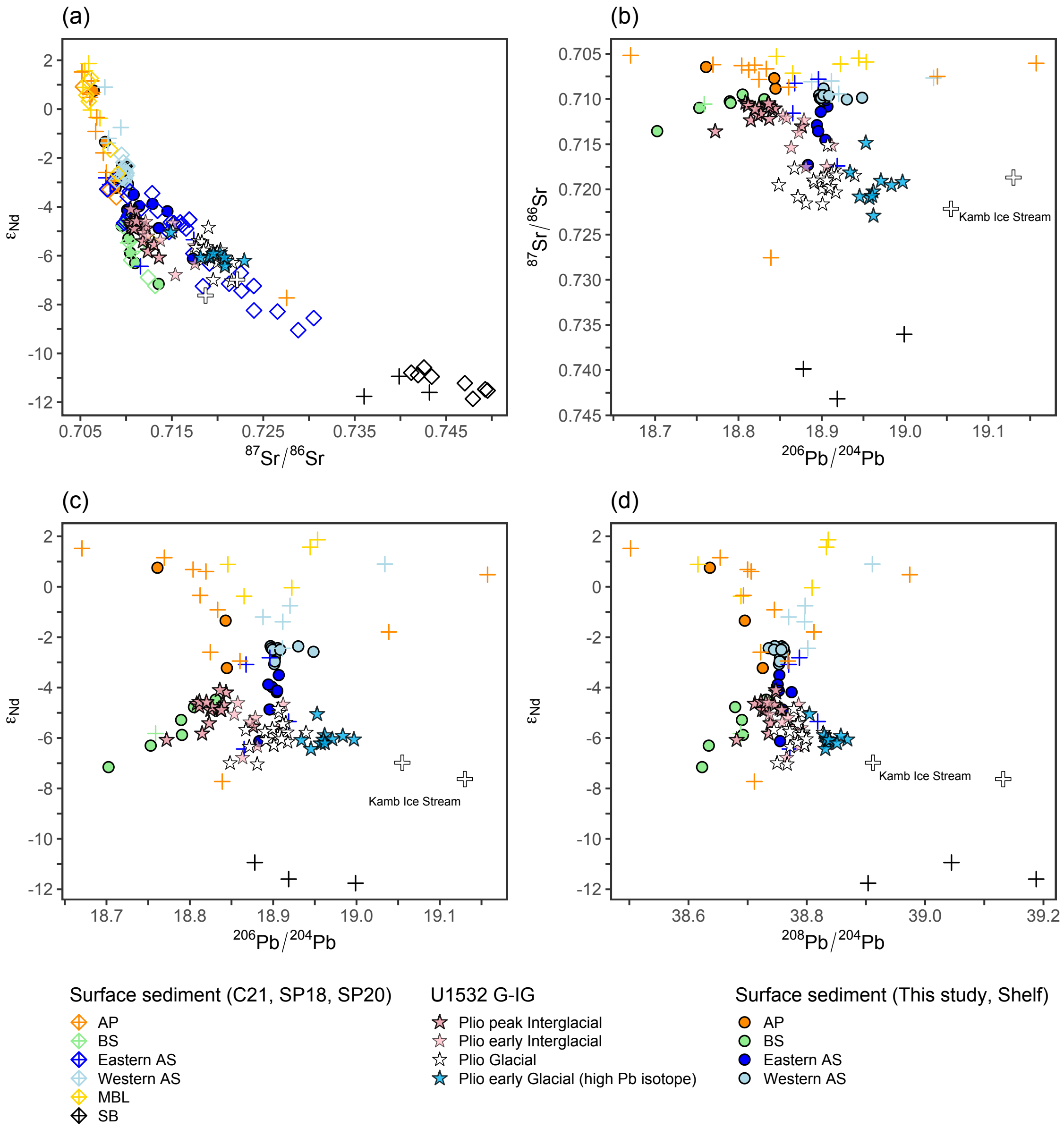

Provenance of modern seafloor surface sediments (shelf) on the West Antarctic margin and Pliocene sediments at Site U1532. Seafloor surface sample data from this study, Simões Pereira et al. (2018) and Simões Pereira et al. (2020) (SP18 and SP20, diamond (9, 51)), Carlson et al. (2021) (C21, cross (52)), and subglacial sediment from Farmer et al. (2006) (open cross (53)) are shown. Pliocene sediment samples from Site U1532 are divided into peak interglacial, early interglacial, glacial, and early glacial (high Pb isotope) samples. Symbols and labelling are the same as in Fig. 3 and *SI Appendix*, Fig. S5. The C21 data from two geographic sectors exhibit distinct isotope ratios in surface sediments: higher $^{206}Pb/^{204}Pb$ and ε_{Nd} values near western Marie Byrd Land (MBL), and higher $^{206}Pb/^{204}Pb$ but distinctly lower ε_{Nd} in the Sulzberger Bay samples. These contrasting isotopic compositions imply limited sediment supply to Site U1532 from the western MBL and Sulzberger Bay sectors throughout the Pliocene.

Fig. S10

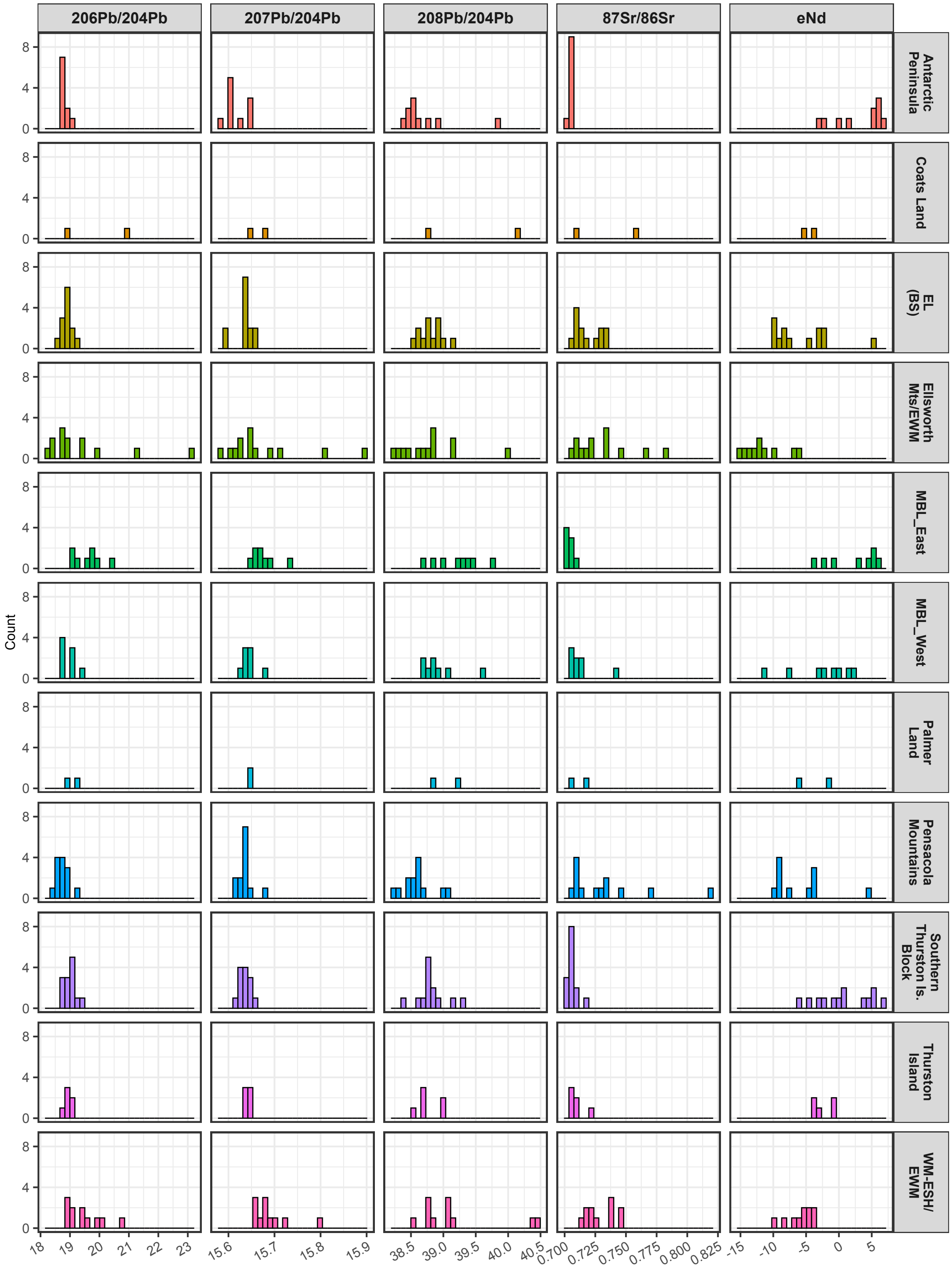

Histogram of isotopic compositions of bedrock samples. Histogram of isotopic compositions of bedrock samples in each West Antarctic sector as well as Coats Land (eastern Weddell Sea,

East Antarctica) and the Pensacola Mountains between East and West Antarctica. BS: Bellingshausen Sea, EL: Ellsworth Land, EWM: Ellsworth-Whitmore Mountains, MBL: Marie Byrd Land, WM–ESH: Whitmore Mountains–Ellsworth Subglacial Highlands (Table S2 and Dataset S3).

Fig. S11

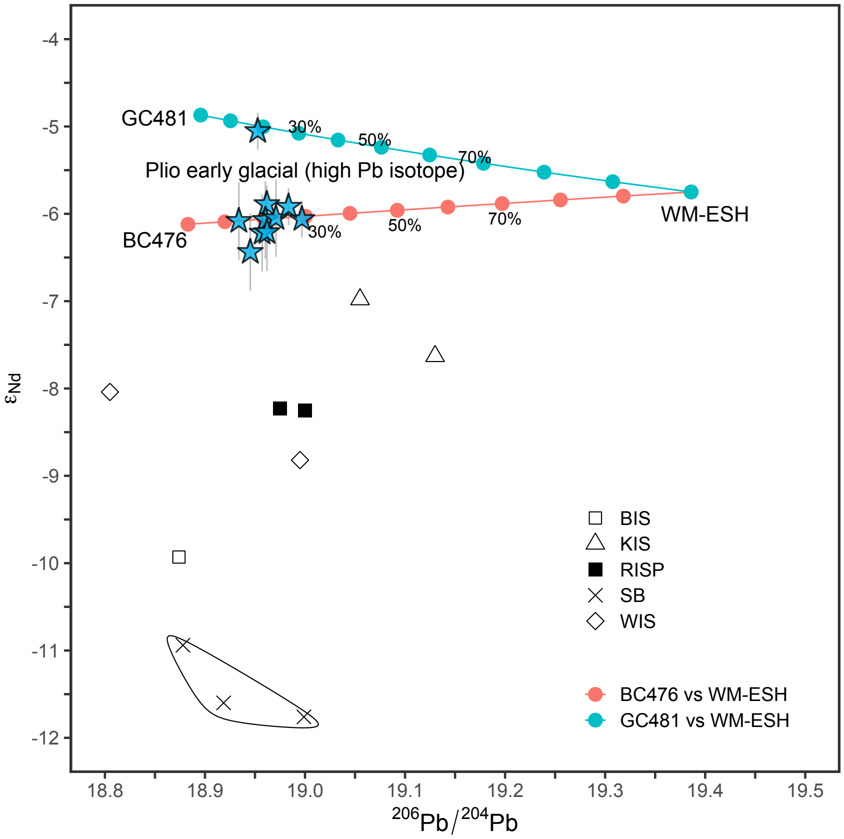

Two-component isotopic mixing curve of Nd and Pb isotopes. Nd and Pb isotope mixing curve between Whitmore Mountains–Ellsworth Subglacial Highlands (WM–ESH) and BC476 (red) or GC481 (cyan) from the eastern Amundsen Sea shelf. U1532 Pliocene early glacial samples with distinct ε_{Nd}–$^{206}Pb/^{204}Pb$ signature (ε_{Nd} below –5 and $^{206}Pb/^{204}Pb$ higher than 18.93) are also plotted (stars). ε_{Nd} data shows 2 s.d. external error bar. Contribution of WM–ESH materials is indicated along the lines in percentage terms. End-member values for BC476 and WM–ESH bedrock samples are described in the main text. End-member values for GC481 are –4.87 ε_{Nd}, $^{206}Pb/^{204}Pb$ = 18.8957, 41.4 µg/g [Nd], and 37.3 µg/g [Pb]. The two-component isotopic mixing equations used in this study are described in Faure and Mensing (2005) (25). The isotopic compositions of modern till underlying the Kamb Ice Stream (KIS), other subglacial sediments beneath Whillans Ice Stream (WIS) and Bindschadler Ice Stream (BIS), and sub-ice shelf sediments recovered by the Ross Ice Shelf Project (RISP) are explained by the mixing of debris eroded from Paleozoic metasedimentary rocks, similar to those exposed in westernmost Marie Byrd Land (and Sulzberger Bay sediments (52), *SI Appendix*, Fig. S9), with detritus derived from Permian-Early Jurassic rocks in the West Antarctic interior, potentially the WM–ESH (53, 61).

Table S1. Pliocene age datums used in this study. Paleomagnetic, radiolarian, and diatom age datums were identified at Site U1532 onboard (1). ID codes of radiolarian and diatom age datums are used to identify these events in *SI Appendix,* Fig. S2. The uncertainty associated with the biostratigraphic age datums are represented by the published range (62) (See *SI Appendix,* Fig. S2).

Datum	Depth 1 (m)	Depth 2 (m)	Age (ka)	Age error	Date type	Note
C2An.2n Base	87.52	87.62	3207	0	paleomagnetism	
C2An.3n Top	100.97	101.09	3330	0	paleomagnetism	
C2An.3n Base	146.36	146.48	3596	0	paleomagnetism	
C3n.1n Top	253.63	253.9	4187	0	paleomagnetism	
C3n.1n Base	283.08	287.63	4300	0	paleomagnetism	
C3n.2n Top	321.65	321.7	4493	0	paleomagnetism	
C3n.2n Base	405.61	405.83	4631	0	paleomagnetism	
C3n.3n Top	422.13	422.43	4799	0	paleomagnetism	
R1<br>LAD *P. titan*	155.99	155.99	3725	275	Rads	3.45-4.0 Ma<br>(published range)
R2<br>LAD *L. coronata*	180.11	180.11	3710	10	Rads	3.7-3.719 Ma<br>(published range)
R3<br>FAD *H. vema*	223.84	223.84	4540	340	Rads	4.2-4.88 Ma<br>(published range)
D4<br>FAD *T. vulnifica*	92.92	92.92	3150	30	Diatom	3-3.4 Ma<br>(published range)
D5<br>FAD *F. interfrigidaria*	156	178.61	4060	130	Diatom	3.7-4.21 Ma<br>(published range)
D6<br>FAD *F. barronii*	224.02	282.91	4400	120	Diatom	4.2-4.6 Ma<br>(published range)
D7<br>FAD *R. costata*	328.14	328.14	4660	80	Diatom	4.58-4.74 Ma<br>(published range)

Table S2. Weighted mean and arithmetic mean Sr-Nd-Pb isotopic compositions for each subregion. One standard deviation is also provided. EL: Ellsworth Land; WM: Whitmore Mountains; ESH: Ellsworth Subglacial Highlands; EWM: Ellsworth-Whitmore Mountains; MBL: Marie Byrd Land.

**Region**	**n**	**$^{87}Sr/^{86}Sr$ Mean**	**$^{87}Sr/^{86}Sr$ SD**	**$^{87}Sr/^{86}Sr$ Weighted mean**	**$^{87}Sr/^{86}Sr$ Weighted SD**	**eNd Mean**	**eNd SD**	**eNd Weighted mean**	**eNd Weighted SD**
Antarctic Peninsula (AP)	10	0.70447	0.00116	0.70460	0.00118	3.09	3.79	0.42	4.20
Coats Land	2	0.73322	0.03400	0.72323	0.02192	-4.55	0.71	-4.65	0.50
EL (BS)	13	0.71817	0.01050	0.71218	0.00922	-5.54	4.44	-5.99	4.12
Southern Thurston Is. Block	14	0.70642	0.00425	0.70533	0.00236	0.74	4.17	0.12	4.57
WM-ESH/EWM	11	0.72968	0.01171	0.72857	0.01031	-5.97	2.08	-5.75	2.00
Ellsworth Mts/EWM	13	0.73059	0.02310	0.71664	0.01121	-11.50	3.05	-11.75	3.21
MBL_East	8	0.70452	0.00202	0.70431	0.00182	2.12	3.91	2.84	2.93
MBL_West	8	0.71306	0.01163	0.70880	0.00670	-2.80	4.66	-3.50	5.00
Palmer Land	2	0.71214	0.00966	0.70611	0.00323	-3.86	3.25	-3.22	2.24
Pensacola Mountains	13	0.73184	0.03228	0.72024	0.01175	-5.92	4.38	-7.33	3.49
Thurston Is.	6	0.70940	0.00583	0.70778	0.00328	-2.24	1.56	-2.41	1.25

Region	n	^{206}Pb/^{204}Pb Mean	^{206}Pb/^{204}Pb SD	^{206}Pb/^{204}Pb Weighted mean	^{206}Pb/^{204}Pb Weighted SD	^{207}Pb/^{204} Pb Mean	^{207}Pb/^{204} Pb SD	207Pb/^{204}Pb Weighted mean	^{207}Pb/^{204}Pb Weighted SD	^{208}Pb/^{204} Pb Mean	^{208}Pb/^{204} Pb SD	^{208}Pb/^{204}Pb Weighted mean	^{208}Pb/^{204}Pb Weighted SD
Antarctic Peninsula (AP)	10	18.8028	0.1308	18.8695	0.1359	15.6172	0.0229	15.6284	0.0235	38.6965	0.4383	38.9193	0.5478
Coats Land	2	19.9369	1.4457	19.7540	1.0364	15.6651	0.0254	15.6619	0.0182	39.4634	0.9993	39.3369	0.7164
EL (BS)	13	18.8871	0.1549	18.8356	0.1189	15.6351	0.0198	15.6381	0.0110	38.7977	0.1738	38.7576	0.1484
Southern Thurston Is. Block	14	19.0043	0.1988	18.9146	0.1699	15.6366	0.0125	15.6406	0.0099	38.8159	0.2200	38.8029	0.2348
WM-ESH/EWM	11	19.4527	0.5886	19.3863	0.4696	15.6906	0.0410	15.6826	0.0274	39.1641	0.6542	39.1296	0.5827
Ellsworth Mts/EWM	13	19.4206	1.3840	18.8545	0.7493	15.6738	0.0868	15.6382	0.0499	38.7913	0.4656	38.7842	0.4144
MBL_East	8	19.5983	0.4725	19.3120	0.3583	15.6756	0.0273	15.6631	0.0188	39.2120	0.3419	39.0205	0.2763
MBL_West	8	18.9618	0.2266	18.9214	0.1721	15.6433	0.0180	15.6393	0.0142	38.9357	0.3016	38.8815	0.2371
Palmer Land	2	19.0349	0.2537	19.0261	0.1840	15.6475	0.0063	15.6473	0.0046	39.0235	0.2613	39.0144	0.1894
Pensacola Mountains	13	18.7594	0.2164	18.6090	0.2171	15.6358	0.0155	15.6334	0.0113	38.5980	0.2436	38.4608	0.2220
Thurston Is.	6	18.9078	0.1263	18.9579	0.0960	15.6428	0.0077	15.6445	0.0068	38.7713	0.1865	38.8413	0.1697

Legends for Datasets S1 to S7

Dataset S1 (separate file). IODP U1532 mean depth-age data generated by the *Undatable* modeling routine (*SI Appendix*, Fig. S2). Uncertainty is also provided.

Dataset S2 (separate file). Strontium (Sr), neodymium (Nd), and lead (Pb) isotopic compositions of detrital fine-grained (< 63 μm) faction of seafloor surface sediments from the West Antarctic margin. AP: Antarctic Peninsula; BS: Bellingshausen Sea; AS: Amundsen Sea.

Dataset S3 (separate file). Sr-Nd-Pb isotopic compositions and major element compositions of bulk bedrock samples in the West Antarctic sector as well as Coats Land and the Pensacola Mountains. Polar Rock Repository ID, rock types, and outcrop locations of bedrock samples are also indicated.

Dataset S4 (separate file). Sr-Nd-Pb isotopic compositions of standard reference materials measured in this study. Previously published reference values are also noted.

Dataset S5 (separate file). IODP Site U1532 Sr-Nd-Pb and coarse-grained fraction results. Classified periods of glacial–interglacial cycles are noted.

Dataset S6 (separate file). Diatom data from IODP Site U1532, used in *Si Appendix*, Fig. S4, with core depth.

Dataset S7 (separate file). Pliocene NGR data from IODP Site U1532, used in Fig. 2, with estimated ages.
