# Supplementary material for: Repeated major inland retreat of Thwaites and Pine Island glaciers (West Antarctica) during the Pliocene
Source: Proc Natl Acad Sci U S A. 2025 Dec 22;123(1):e2508341122. doi: 10.1073/pnas.2508341122 (PMC12773727; doi:10.1073/pnas.2508341122)
Supplement: Supplementary file 9 — Dataset S08 (DOCX) [file pnas.2508341122.sd08.docx]

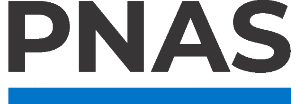


**Expedition 379 Science Party**

The following authors were part of the Expedition 379 Science Party:

Karsten Gohl^h^, Julia S. Wellner^i^, Adam Klaus^m^, Denise Kulhanek^m,n^, Thorsten Bauersachs^o^, Steven M. Bohaty^o^, Margot Courtillat^p^, Ellen A. Cowan^f^, Marcelo A. De Lira Mota^q^, Mariana S.R. Esteves^r^, John M. Fegyveresi^s^, Thomas Frederichs^t^, Liang Gao^u^, Anna Ruth Halberstadt^e^, Claus-Dieter Hillenbrand^c^, Masao Iwai^b^, Ji-Hoon Kim^v^, Theresa M. King^w^, Johann P. Klages^h^, Sandra Passchier^x^, Michelle L. Penkrot^g^, Joseph G. Prebble^y^, Waliur Rahaman^z^, Benedict T.I. Reinardy^aa^, Johan Renaudie^bb^, Delaney E. Robinson^i^, Reed P. Scherer^cc^, Christine S. Siddoway^d^, Li Wu^dd^ & Masako Yamane^ee^

^m^IODP Texas A&M University, USA. ^n^Kiel University, Institute of Geoscience, Germany. ^o^Institute of Earth Sciences, University of Heidelberg, Germany. ^p^Centre of Education and Research on Mediterranean Environments Laboratory, University of Perpignan, France. ^q^Institute of Geosciences, University of São Paulo, Brazil. ^r^Centre for Arctic Gas Hydrate, Environment and Climate (CAGE), The Arctic University of Norway, Norway. ^s^School of Earth and Sustainability, Northern Arizona University, USA. ^t^MARUM – Center for Marine Environmental Sciences, University of Bremen, Germany. ^u^School of the Earth Sciences and Resources, China University of Geosciences, China. ^v^Korea Institute of Geoscience & Mineral Resources (KIGAM), Republic of Korea. ^w^College of Marine Science, University of South Florida, USA. ^x^Earth and Environmental Studies, Montclair State University, USA. ^y^Paleontology Department, GNS Science, New Zealand. ^z^National Center for Polar & Ocean Research (NCPOR), India. ^aa^Dept. Sustainable Development, Environmental Science and Engineering (SEED), KTH Royal Institute of Technology, Sweden. ^bb^Museum fur Naturkunde, Leibniz-Institut fur Evolutions und Biodiversitätsforschung, Germany. ^cc^Geology and Environmental Geosciences, Northern Illinois University, USA. ^dd^School of Geographic Sciences, East China Normal University, China. ^ee^Institute for Space-Earth Environmental Research, Nagoya University, Japan.

**Author Contributions:** IODP Expedition 379 Science Party members contributed to sample collection and participated in generating shipboard data.
